# Novel enzymatic route to the synthesis of C-8 hydroxyflavonoids including flavonols and isoflavones

**DOI:** 10.1038/s41598-024-68513-5

**Published:** 2024-08-06

**Authors:** Kinga Dulak, Sandra Sordon, Agata Matera, Aleksandra Wilczak, Ewa Huszcza, Jarosław Popłoński

**Affiliations:** 1 Department of Food Chemistry and Biocatalysis, Wroclaw University of Environmental and Life Sciences, Wroclaw, Poland; 2 Hirszfeld Institute of Immunology and Experimental Therapy, Polish Academy of Sciences, Wroclaw, Poland

**Keywords:** Biocatalysis, Biotransformation, Monooxygenase, FMO, Flavonoids, Hydroxylation, *Ortho*-hydroxylation, 8-Hydroxyquercetin, Gossypetin, Recombinant protein, Synthetic biology, Biocatalysis, Biocatalysis, Enzymes, Enzymes

## Abstract

Flavin-dependent monooxygenases (FMOs) are a valuable group of biocatalysts that can regioselectively introduce a hydroxy group for the targeted modification of biologically active compounds. Here, we present the fdeE, the FMO from *Herbaspirillum seropedicae* SmR1 that is a part of the naringenin degradation pathway and is active towards a wide range of flavonoids—flavanones, flavones, isoflavones, and flavonols. Bioinformatics and biochemical analysis revealed a high similarity between the analyzed enzyme and other F8H FMOs what might indicate convergent evolutionary mechanism of flavonoid degradation pathway emergence by microorganism. A simple approach with the manipulation of the reaction environment allowed the stable formation of hydroxylation products, which showed very high reactivity in both in vivo and in vitro assays. This approach resulted in an 8-hydroxyquercetin—gossypetin titer of 0.16 g/L and additionally it is a first report of production of this compound.

## Introduction

Natural products from plants and other biological sources remain an undiminished source of new pharmaceuticals or their precursors^1^. The biological activity of the plant secondary metabolites such as flavonoids e.g., quercetin, resveratrol, or catechins has been documented^2^, along with their ability to potentiate the activity of various anticancer drugs^3^. The presence of a phenolic hydroxyl group and conjugated double bonds gives quercetin strong antioxidant activity, closely linked to the prevention and treatment of cardiovascular disease and cancer^4^. The synthesis of 8-hydroxyquercetin (gossypetin) has been studied since the first half of the twentieth century^5^. In recent years, this derivative has been described by many researchers due to its wide range of properties. The literature has primarily highlighted medical aspects as therapeutic regimes in osteosarcoma, with potential implications in the prevention of metastasis^6^, and a novel MKK3 and MKK6 inhibitor and perhaps a practical method in chemoprevention of esophageal cancer^7^. In addition, due to its AMPK activity, which is the highest among flavonoids, it may serve as a potential candidate in the treatment of diabetes and its related complications^8^. Furthermore, it inhibits ox-LDL uptake and the formation of lipid-laden foam cells while controlling the balance of cholesterol transport. Thus, gossypetin acts against ox-LDL by activating PPARα and/or depressin PPARγ, which in turn simulates cholesterol removal and calcification from macrophages and delay atherosclerosis^9^. However, its utility does not end there, it can be applied as a taxonomic marker of plants at the generic and subfamilial level^10^.

The position and degree of hydroxylation are crucial in determining their antioxidant activity^11^, especially the formation of the C7–C8 catechol motif is closely linked to valuable health-promoting properties^12–16^. Hydroxylation is one of the main modifications that have a profound effect on the physical and biological activities of flavonoids^17^. However, selective and efficient hydroxylation of aromatic compounds using classical synthetic chemistry is hardly feasible and requires toxic reagents^18^. Despite the reported progress in the use of hydrogen peroxide and metal catalysts, i.e. vanadium, palladium, TiO_2_, or chemical oxidation in supercritical carbon dioxide, the selectivity of these methods is still limited. Therefore, complex, multi-step processes such as the Hock process are still mainly used to obtain hydroxylated aromatics^19^. Enzyme catalysis, is an excellent alternative, although most of the identified enzymes belong to a cytochrome P-450 monooxygenases that depend on compatible reductase^20^, leak sufficient regioselectivity, or their yields are very low, therefore limiting possible application. In turn, the Rieske oxygenases present among the bacteria were found for applications in the production of homochiral starting materials and bioremediation from aromatic contaminants. However, the direct application of those enzymes is hindered by the sensitivity of the [2Fe-2S] cluster to oxygen^21^. Copper oxidases characterize low selectivity, many side products are generated as a result—chinone formation, and moreover, some flavonoids act as their inhibitors^22^. On the other hand, flavoprotein monooxygenases (FMOs), which belong to group B of flavin-dependent monooxygenases, can directly use NADPH as an electron donor^23^, exhibit excellent regioselectivity, but until now we had limited data regarding FMOs active on flavonoids^24^ and specifically responsible for the formation of C7–C8 catechol moiety.

Beyond our previous report regarding the identification of F8H from *Rhodotorula glutinis* KCh735^25^, there is enzyme previously reported as being responsible for the first step in the naringenin degradation pathway—fdeE from *Herbaspirillum seropedicae* SmR1^26,27^ although not characterized yet in terms of biocatalytic application. Sequencing of the *H. seropedicae* SmR1 genome revealed the presence of the fde operon, involved in metabolism of aromatic compounds^28^. Researchers proposed two pathways for the degradation of naringenin by *H. seropedicae* SmR1: one involving C-ring opening and clavage resulting in two aromatic compounds, and another requiring initial C8 carbon hydroxylation and further oxidation of ring A. Both pathways lead to intermediate compounds, ultimately degraded to protocatechuate by enzymes form the fde operon. Those compounds are further metabolized by the pca operon. Other enzymes from the fde operon may contribute to the meta cleavage pathway, producing intermediates for the tricarboxylic acid (TCA) cycle^27^. This might suggest, that other similar degradation pathways might evolved independently in microbial communities to degrade common flavonoid compounds like naringenin.

Application of microorganisms or isolated enzymes as biocatalysts is a well-established strategy for the synthesis of natural compounds with high added value, although the future of natural compound synthesis lies in synthetic biology where implementation of novel activities to existing and optimized biosynthetic pathways may lead to the production of new compounds starting from simple carbon sources^29–31^. Our research focuses on the identification of novel activities that can be further applied in flavonoid microbial biosynthesis. In addition, this approach provides environmentally friendly and efficient production of compounds that until recently were beyond the reach of science.

Herein, we report the biochemical characterization of fdeE from *H. seropedicae* SmR1 and results indicate activity with broad spectrum of flavonoid substrates. Moreover, this is the first report of selective *ortho*-hydroxylation of flavonols and isoflavones at the C-8 position by FMO and the first report of the production of 8-hydroxyquercetin—gossypetin, achieved by a simple strategy to stabilize the resulting products in vitro, which upon optimization can be widely applied to large-scale production of these compounds.

## Materials and methods

### Strains

For cloning and maintenance *E. coli* DH5α and for expression *E. coli* BL21 (DE3) strains were used (New England Biolabs Inc., Ipswich, USA).

### Chemicals

The compounds and media were purchased from Sigma-Aldrich (St Louis, USA), Merck Millipore (Burlington, Massachusetts, USA), or Carbosynth (Compton, Berkshire, UK). Quercetin and other substrates were purchased from Carbosynth (Compton, Berkshire, UK). The antibiotics were purchased from Cayman Chemical Company (Ann Arbor, Michigan, USA). Restriction enzymes (*Bsa*I-HFv2, *Bbs*I-HF), T4 DNA ligase, *Taq* PCR Kit, Plasmid Miniprep Kit, and all other necessary reagents for molecular biology were purchased from New England Biolabs Inc. (Ipswich, USA). The UPLC, LC–MS grade solvents, and purification eluents used in this work were purchased from Merck KGaA (Darmstadt, Germany). Sephadex LH-20 Gel used for product purification was purchased from Sigma-Aldrich (St Louis, USA).

### Alignments and modeling

All pairwise and multiple amino acid sequence matches were performed with the Clustal Omega algorithm (https://www.ebi.ac.uk/Tools/msa/clustalo/) with standard parameters visualization performed using iTOL web-based program^32^. GenBank IDs of the used amino acid sequences are shown in Supplementary Table S1. A detailed comparison of the fdeE with RgF8H^25^ was made, based on their significant homology. The analysis focused on the search for characteristic monooxygenase motifs: the FAD binding motif (GxGxxG), the F motif (FxGxxxHxxxy), and the GD motif (GDAxHxxxPxxxxG)^33^.

All molecular docking experiments were performed using the UCSF Chimera 1.16 software^34^ and AutoDock Vina 1.1.2^35^. The 3D models of fdeE, LjF8H, and RgF8H were constructed using AlphaFold^36^ and obtained directly from the UniProt database (D8J0W9, A0A830U7D1, A0A109FFD3). In the case of RgF8H, a homologous structure was used with 99% amino acid identity. The differences between the residues of the model used and the exact RgF8H sequence are > 12 Å away from the FAD and substrate binding residues. Docking of the FAD cofactor to all three models was performed using aligned structures of RgF8H and LfF8H to fdeE within the AutoDockVina Volume box covering the whole proteins. Docking of the substrates (naringenin, genistein and quercetin) to RgF8H and fdeE was carried out in the cavity adjacent to the FAD binding site, with the AutoDockVina Volume box for both structures set to xyz: 13.0823, − 1.4892, − 7.05058; size: 19.602, 21.413, 26.101. All selected docking positions were based on the highest score and did not clash or come into contact with the FAD cofactor.

### Cloning and expression of hydroxylases

Codon-optimized sequences (ORFs) encoding protein flanked by *Bsa*I restriction sites were inserted into pRhaBAD_12 vector^37^ according to the Golden Standard Modular Cloning Strategy (GS MoClo)^38^. ORFs encoding codon-optimized protein were also cloned together with promoter pJ23100 (http://parts.igem.org/Promoters/Catalog/Anderson), T7 RBS, and terminator T7 into pSEVA23g19g1 vector. A summary of all cloning details including all primer sequences, corresponding vectors, and strains in which they were used are presented in Supplementary Table S2. All genetic constructs were verified by sequencing (Macrogen Europe). Transformed *E. coli* BL21 (DE3) strains were cultured in 500 mL of liquid LB medium (1% tryptone, 0.5% yeast extract, 0.5% NaCl; m/m) supplemented with kanamycin (30 µg/mL). The cultures were initially incubated at 37 °C, 120 rpm until OD_600_ reached 0.6. The cells were then induced by adding 15 mM rhamnose (pRhaBAD_12) and riboflavin (8 µg/mL). Protein expression was performed at 30 °C for 14 h.

### Protein purification

The bacterial cell pellet obtained after expression of the fdeE (~ 3 g wet weight from 500 mL culture) was washed twice with sodium phosphate buffer (pH 7.5, 50 mM). After disruption (incubation with lysozyme—300 μg/mL, at 4 °C for 1 h and sonication on ice for 2.5 min by the following procedure—5 s pulses and 5 s pauses at 80% amplitude using Vibra-Cell Ultrasonic Liquid Processor VCX 130 (Sonics & Materials, Inc., Newtown, USA), the cell lysate was centrifuged (14,000×*g*, 20 min), and the supernatant was passed through a 0.45 μm PEEK filter before IMAC chromatography. Chromatography was performed using a pump system (Watson-Marlow, Cornwall, UK). The filtered cell extract was applied to a 5-mL IMAC HisTrap™ HP column (GE Healthcare), following the standard purification procedure using sodium phosphate buffer (pH 7.5, 20 mM, containing 0.5 M NaCl, and 20 mM imidazole for binding and 500 mM for elution). Fractions with the recombinant protein were combined and desalted by gel chromatography using PD-10 Desalting Columns (GE Healthcare) with 20 mM phosphate buffer, pH 7.5 (or MiliQ water—for ThermoFAD assay). Purified enzyme fractions were stored at 4 °C or used immediately after purification. Protein concentration was determined after desalting by BCA assay^39^ using a spectrophotometer (Eppendorf BioSpectrometer Kinetic) and bovine serum albumin as a reference for the calibration curve.

### Biochemical characterization of enzymes

The oxidation of NADPH or NADH (1 mM) was monitored in an in vitro reaction with and without substrate (naringenin 1 mM) using a spectrophotometer (Eppendorf BioSpectrometer Kinetic) at λ = 340 nm. To investigate the effect of flavin adenine dinucleotide (FAD) on enzyme activity, FAD was added to the standard reaction mixture at a concentration of 0.01 mM and the reaction was run for 30 min.

The standard in vitro activity assay (0.01 mM naringenin, 0.1 mM NADPH, 100 μL crude or 20 μL purified protein extract (2.0 mg/mL)) was carried out in 25 mM sodium phosphate buffer (pH 7.5) at 30 °C, 800 rpm for 15 min. Reactions were stopped by the addition of an equal volume of methanol, vortexed, centrifuged (21,000×*g*, 5 min), evaporated, dissolved in 1 mL of methanol, filtrated and 200 µL of the alcoholic solution was analyzed directly by UPLC-DAD. A culture of an *E. coli* strain without plasmid was used as a control.

The second approach involved an in vivo assay, transformed *E. coli* cells expressing recombinant hydroxylase were cultivated aerobically at 37 °C, 120 rpm in 0.6 or 30 mL LB medium supplemented with kanamycin (30 µg/mL). Substrates were added to a concentration of 0.01 mM as a dimethyl sulfoxide (DMSO) stock solution (1 mM) after a culture reached the optimal density at OD_600_ = 0.6 and shaken 16 h at 30 °C, 120 rpm. 0.2 mL of ethyl acetate was added to 0.6 mL of the reactions mixture, vortexed, centrifuged (21,300×*g*, 5 min) and 50 µL of an organic fraction was transferred to 0.45 mL of methanol, filtrated, and analyzed by UPLC-DAD.

To ensure the best conditions for hydroxylase activity, optimum temperature (10–50 °C), pH (4.0–10.0), ionic strength (0–1.5 M), buffer molarity (5–100 mM), co-solvent addition (0–30%) and ionic strength (0–1.5 M) were analyzed photometrically using a microplate reader (BioTEK, SYNERGY H1, Biokom, Poland) on a basis of NADPH consumption, reactions (100 µL) were stopped by addition of 100 µL of methanol. The stability of the enzyme was determined using the ThermoFAD method^40^. Samples contained 10 µL (2.0 mg/mL) of the purified enzyme and 15 µL of 50 mM buffers with a pH range of 3.5–10.0. Reaction mixtures were prepared in 96-well plates and assayed using the Real-Time PCR Detection System according to the CFX96 Touch Protein Thermal Shift Assay Protocol manual^41^.

### Characterization of substrate specificity

All compounds used to determine substrate specificity are gathered in Supplementary Table S3. Reactions were carried out in phosphate buffer (pH 7.5, 25 mM) according to the procedure described in the previous section, with a final substrate concentration of 0.05 mM. Flavonoid conversions (Table 1b) were monitored by UPLC-DAD liquid chromatography and LC–MS analysis after 10 and 30 min. Specific activity was calculated as units per milligram of protein, and one unit (U) of activity was defined as the amount of enzyme that converts 1 μmol of substrate per 1 min.

**Table 1:** (a) Summary of the analysis of the biochemical properties of fdeE in vitro. Data in all panels represent averages over three replicates. Stability of fdeE analyzed via ThermoFAD under different conditions. (b) Substrate specificity of fdeE, specific activity [U—one unit defined as 1 µmol of substrate converted per 1 min by 1 mg of enzyme]. UPLC-DAD, ESI/MS analysis, and UV maxima of substrates and products.

(a)													
Factor	Enzyme activity												
Temperature	30 ℃												
Ionic strength	0–300 mM												
Solvent addition	< 10%												
Buffer molarity	5–10 mM												
pH	5.0												

Factor	Enzyme stability												
Temperature	20–40 ℃												
pH	7.5 (Phosphatate buffer) 8.0 (Tris–HCl buffer)												

(b)													
Substrates							Products						
Name	Structure	UPLC (t_R_) [min]	Calculated mass	Mass		UV maxima [nm]	Structure	UPLC (t_R_) [min]	Calculated mass	Mass		UV maxima [nm]	Specific activity [U/mg]
				[M + H]^+^ m/z^+^	[M-H]^−^ m/z^−^					[M + H]^+^ m/z^+^	[M-H]^−^ m/z^−^		
Naringenin		3.237	272.25	273	271	288		2.748	288.25	289	287	294	38 ± 0.73
Hesperetin		3.185	302.28	303	301	287		2.741	318.28	319	317	291	38 ± 1.24
7-Hydroxyflavanone		3.154	240.25	241	239	275		2.878	256.25	257	255	292	45 ± 1.92
Pinocembrin		3.590	256.25	257	255	289		3.081	272.25	273	271	294	27 ± 0.56
Eriodictyol		2.961	288.25	289	287	287		2.493	304.25	305	303	292	35 ± 1.12
Chrysin		3.766	254.24	255	253	267		3.267	270.24	271	269	279	27 ± 0.98
Baicalein		3.283	270.24	271	269	275		2.866	286.24	289	287	285	6 ± 0.03
Diosmetin		3.390	300.26	301	299	251, 343		2.948	316.26	317	315	279, 335	25 ± 0.31
Apigenin		3.515	270.24	271	269	267, 334		3.021	286.24	287	285	280, 303	41 ± 1.43
Luteolin		3.221	286.24	287	285	252, 345		2.769	302.24	303	301	279, 337	16 ± 0.82
4′,7-Dihydroxyflavone		3.085	254.24	255	253	328		2.682	270.24	271	269	265, 326	5 ± 0.26
Fisetin		2.909	286.24	287	285	247, 359		2.533	302.24	303	301	254, 363	22 ± 0.15
Quercetin		3.226	302.24	303	301	255, 371		2.777	318.24	319	317	259, 338	23 ± 1.67
Myricetin		2.891	318.24	319	317	252, 373		2.482	334.23	335	333	257, 340	21 ± 1.98
Morin		3.546	302.24	303	301	264, 365		3.093	318.24	319	317	254, 353	46 ± 0.72
Genistein		3.401	270.24	271	269	260		2.840	286.24	289	287	266	8 ± 0.53
Biochanin A		3.755	284.26	285	283	260		3.252	300.26	301	299	267	2 ± 0.09

### Formation rate of hydroxyflavonoids

On the basis of the high reactivity of formed products and possible analytical bias we used an internal standard (IS)—dibenzofuran verified as non-substrate and non-inhibitor (data not shown). The first test involved an in vivo reaction in *E. coli* DH5α cells under aerobic conditions in 30 mL of LB medium supplemented with kanamycin (30 mg/mL) at 120 rpm and 30 °C with 10% overnight (ON) inoculum (v/v) and a final total concentration of flavonoids and IS of 1 mM and 0.1 mM, respectively, supplemented to the reaction mixture from a DMSO stock solution. Samples (600 µL) were collected at 0 and after 2, 4, 6, 8, and 24 h, and extracted with 200 µL of ethyl acetate. 50 µL of the organic fraction was mixed with 450 µL of methanol, filtrated, and analyzed by UPLC-DAD.

The second approach involved an in vitro assay, the reaction was carried out in 1 mL of a mixture containing 0.1 mM flavonoids, 1 mM NADPH, 0.01 mM IS, and 100 µL crude protein. Samples were incubated at 30 °C at 800 rpm. The control reactions contained crude protein thermodenatured at 95 °C for 30 min. Samples were collected after 5, 15, 30, and 60 min of reaction and 100 µL of methanol was added to an equal volume of the reaction mixture, vortexed, centrifuged, filtrated and, analyzed directly by UPLC-DAD. Naringenin, luteolin, chrysin, apigenin, and quercetin were used for in vivo assays, while naringenin, luteolin, and quercetin were used as substrates for in vitro assays. Two C-8 FMOs were used for both approaches—fdeE and RgF8H (Supplementary Table S2).

### Hydroxyderivatives stabilization

To increase the stability of the hydroxylation products, compounds with reducing properties were used: dithiothreitol, cysteine, thiosulphate, and sodium dithionite. The reaction mixture (5 µL enzyme (2 mg/mL), 0.025 mM substrate, 2 mM NADPH, 5, 25 or 100 mM reducing agent, in 1 mL of 25 mM sodium phosphate buffer, pH 7.5) was run, sampled, and analyzed according to the procedure described in the previous section.

### Kinetic parameters

Kinetic parameters were determined for the same five substrates: naringenin, luteolin, apigenin, quercetin and chrysin. The reaction mixture (30 µL enzyme (1.8 mg/mL), 0.05 mM substrate, 1 mM NADPH, 25 mM DTT, 50 mM glucose, 100 µL GDH (20 mg/mL), in 1 mL of 25 mM sodium phosphate buffer, pH 7.5) was run, sampled for 30 min at 2.5 min intervals and analyzed according to the procedure described in the previous section.

### Purification of hydroxyquercetin

Semi-preparative reactions were carried out with crude enzyme extracts in a total volume of 100 mL in a 250-mL baffled flask (30 °C, 120 rpm) for 24 h. The reaction was carried out in 25 mM phosphate buffer (pH 7.5) containing 0.5 mM IS, 25 mM DTT, 0.1 mM NADPH, 0.1 mM NADP^+^, 1 mL GDH (20 mg/mL lyophilized pure enzyme), 5% glucose, 5% (v/v) DMSO and 100 mg quercetin (3.3 mM). Reaction samples (600 μL) were taken over time to control the reaction progress. Additional portions of glucose (10 mg) to enhance the cofactor regeneration system was added after 7 and 21 h. After 24 h, samples were divided into 3 fractions (30 mL each) and extracted by three different methods: three times with 5 mL of ethyl acetate or 1-butanol or methanol of the previously lyophilized whole fraction and subsequent filtering of the extract. The organic fractions from the first two techniques were evaporated to dryness, resuspended in 3–5 mL of methanol, and purified on a Sephadex LH-20 Gel with methanol as eluent. The composition of the collected fractions was continuously monitored using UPLC-DAD. The fractions containing the quercetin hydroxylation product were evaporated, weighed, dissolved in DMSO-*d*_*6*_, and subjected to nuclear magnetic resonance (NMR) spectroscopy.

### Chemical analysis

UPLC-DAD and LC–MS analysis was performed according to the procedure described by us previously^25^. NMR spectra (^1^H-NMR, ^13^C-NMR, ^1^H-^1^H NMR (COSY), and ^1^H-^13^C NMR (HSQC, HMBC)) were recorded on a DRX Bruker Avance TM 600 (600 MHz) instrument in DMSO-*d*_*6*_.

## Results

**Figure 1:**
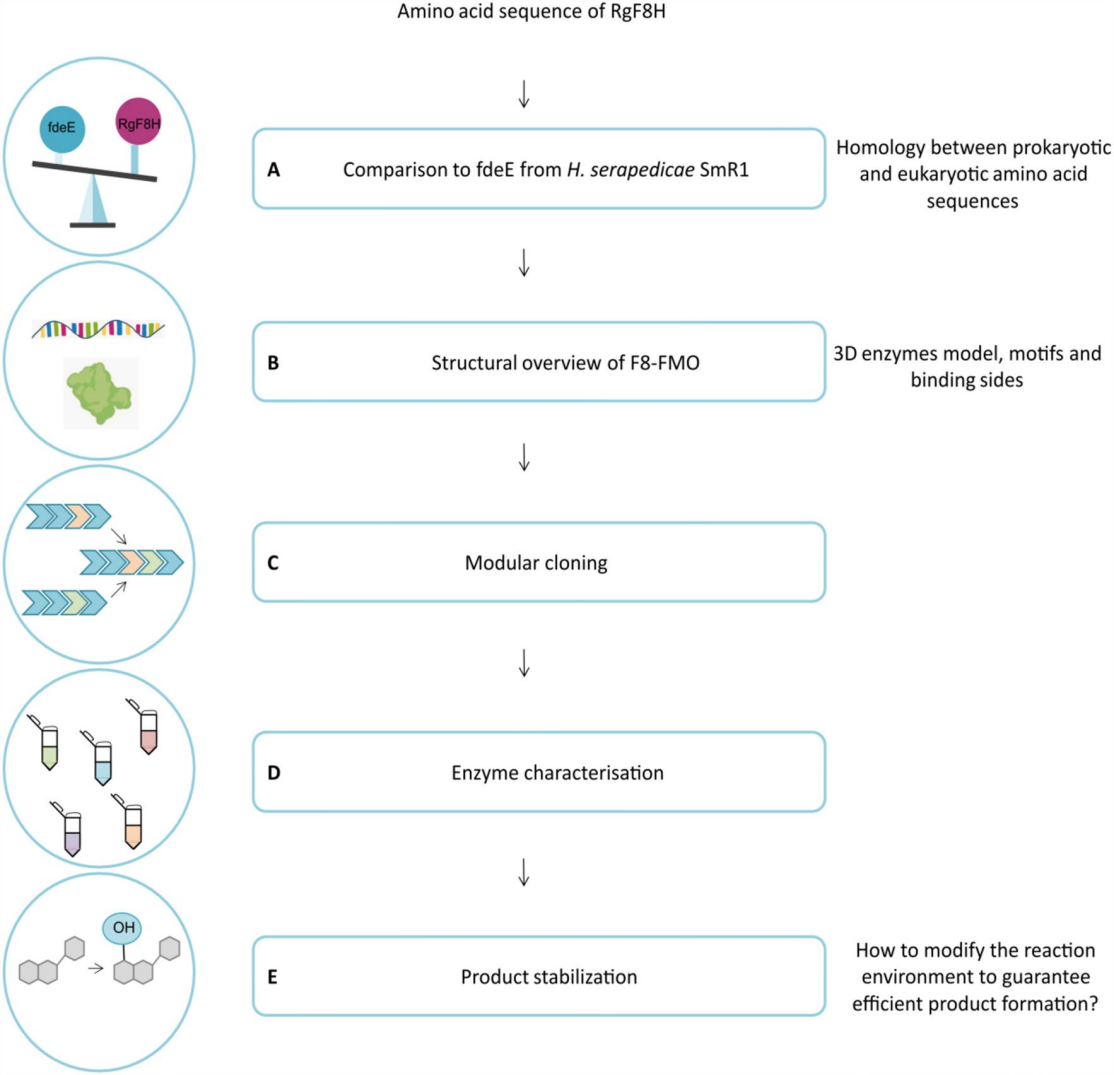
Flow scheme of the approach for the characterization of hydroxylase from *H. serapedicae* SmR1 and obtaining of hydroxy product, with steps A–E.

Bering in mind the homology between the hydroxylase from *H. serapedicae* SmR1 (fdeE^27^) and the already described by us enzyme from *R. glutinis* KCh 735 (RgF8H^25^) in the first step we compared the amino acid sequences of the two enzymes (step A, see Fig. 1 for a diagram of the steps). We carefully analyzed the structural information of related enzymes and explored important motifs and suggestions for their use (step B). Further, the protein-coding sequence that meets the prediction criteria was cloned from synthetic genes and expressed in *E. coli* cells (step C). The investigated protein was produced, purified, and biochemically analyzed towards hydroxylase activity and regioselectivity (step D). The final step of the study focused on the stabilization of the resulting readily oxidizable products (step E).

**Figure 2:**
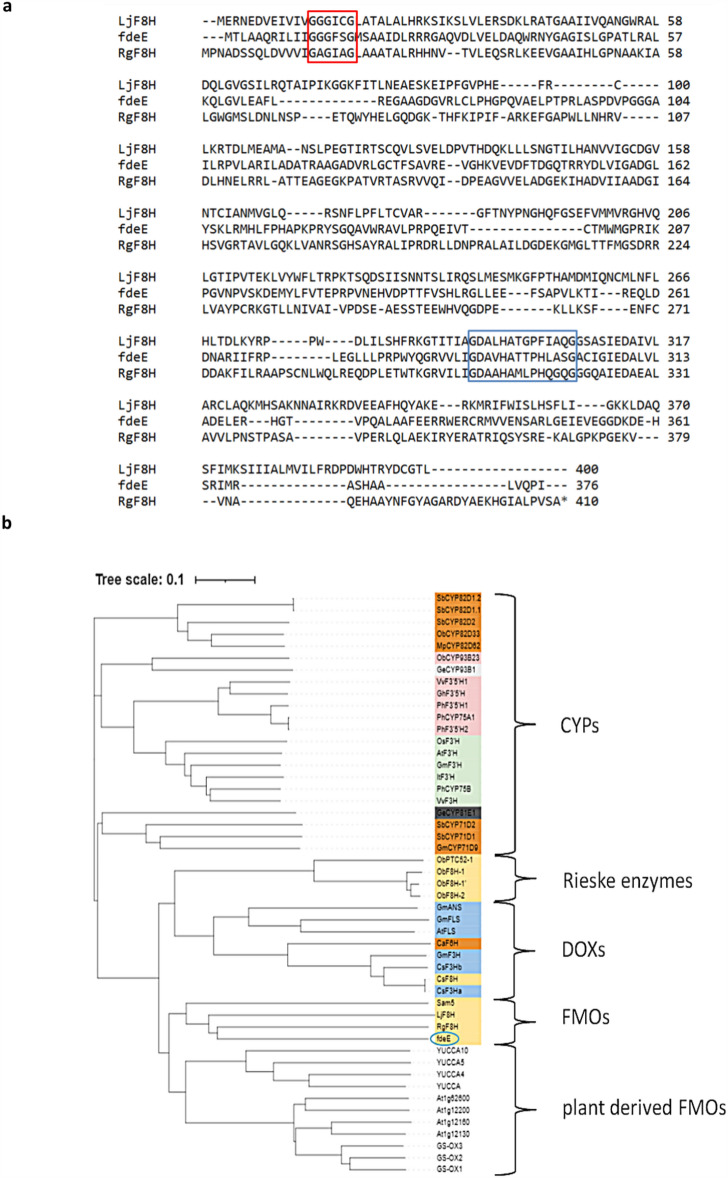

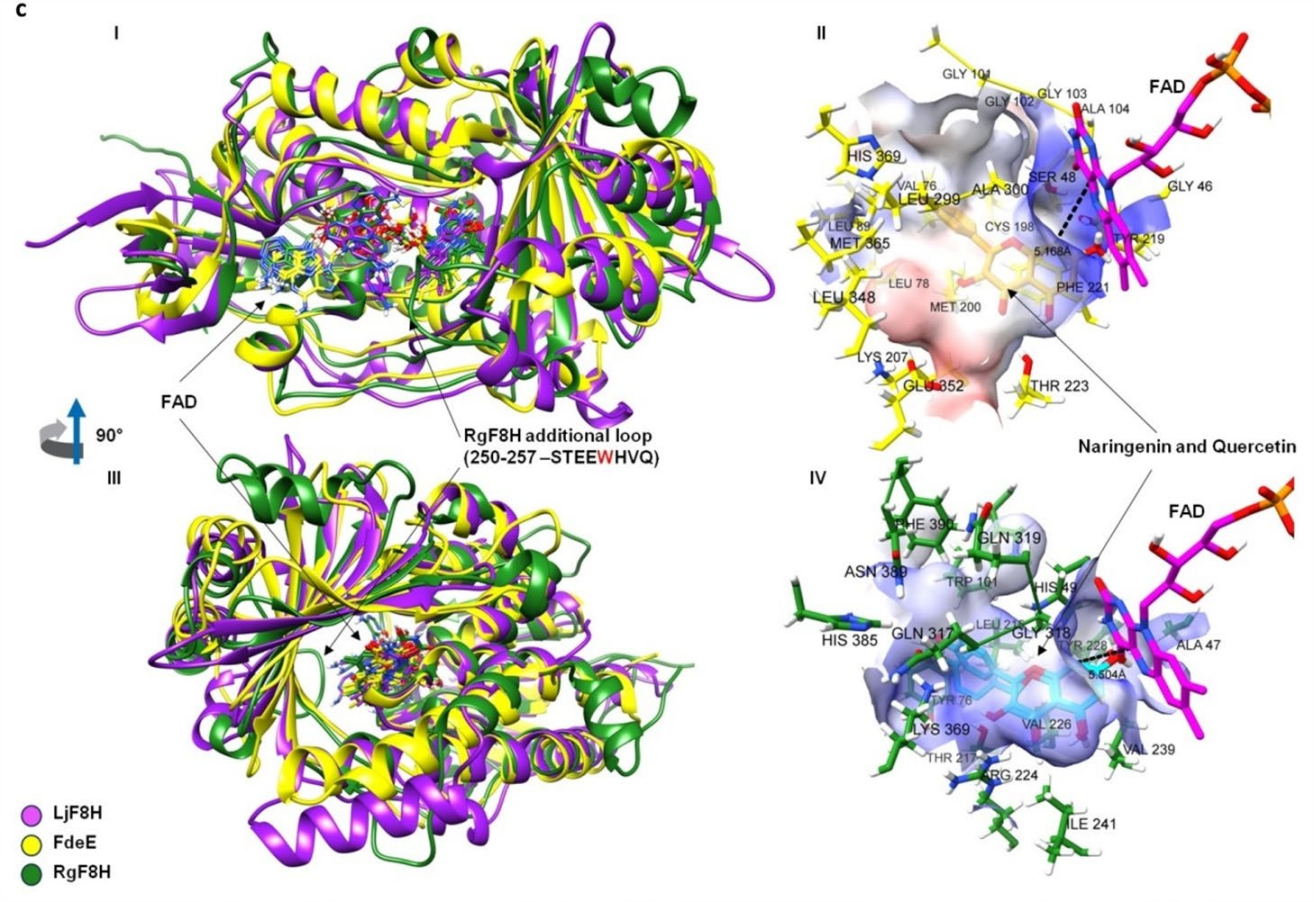
(**a**) Multiple sequences alignment encoding the amino acids: LjF8H—line 1, fdeE—line 2, and RgF8H—line 3. Highly conserved FAD and GD motifs were highlighted in boxes using red and blue underscores, respectively. (**b**) Phylogenetic analysis based on FMOs, CYPs, Rieske-type, plant derived FMOs, and DOXs sequences. Protein sequences downloaded from the NCBI were aligned using Clustal Omega software (https://www.ebi.ac.uk/Tools/msa/clustalo). The cladogram was constructed using iTOL software (https://itol.embl.de/). Labels used: yellow colour—C-8 hydroxylases, blue colour – C-3 hydroxylases, orange colour—C-6 hydroxylases, green colour—C-3’ hydroxylases, pink colour—C-3′5’ hydroxylases, light grey colour—C-2’ hydroxylase, and dark grey colour—C-2 hydroxylase. Accession numbers of amino acid sequences used are available in the Supplementary material (Table S1). (**c**) I, III: Aligned predicted 3D protein structures of fdeE, LjF8H, and RgF8H with many poses of the docked FAD cofactor. II, IV: Simplified representation of part of the structure of the II-fdeE or IV-RgF8H with FAD,naringenin and quercetin docked (highest score hits), residues that are identical in both proteins are omitted in the graphic, surfaces were colored according to the electrostatic potential. The models and docking experiments were made in UCSF Chimera and figure created in ChimeraX 1.6.1.

A blast search to date described F8H, RgF8H^25^ and LjF8H^33^ revealed significant homology to fdeE, with coverage rates of 53% and 81%, respectively. The next step was to analyze FMO-specific binding motifs. RgF8H, LjF8H, and fdeE contain a Rossmann fold motifs (GxGxxG) and a GD motif (GDAxHxxxPxxxxG). Interestingly, despite sharing the same activity, all these enzymes lack the characteristic motifs, suggested to connect the FAD and NAD(P)H binding sites (FxGxxxHxxx)^25,33,42^ in their structures, despite that activity is detected. It is speculated that this motif is necessary for the regeneration of the FAD without a redox protein partner (Fig. 2a), which might suggest that the three enzymes belong to the same new FMO subclass or share different NADPH-binding domains.

A cladogram was constructed among known flavonoid hydroxylases (Fig. 2b). The sequence of fdeE, was grouped within F8H, a member of the FMO family, and shows the highest homology with RgF8H^25^ (query cover—53%, percent identity—29.26%), and LjF8H (query cover—81%, percent identity—25.69%). Aligned structures of the 3D models of fdeE, LjF8H, and RgF8H indicate that all three enzymes share a similar overall architecture, with 10–15% of residues being identical in the RR distance map. All proteins dock FAD in the same cavity, where isoalloxazine indicates substrate binding pockets with a tunnel responsible for high regioselectivity of the reaction. However, in the case of RgF8H, an additional loop (residues 250–257) with W254 facing FAD packs it more tightly than in the other two enzymes (Fig. 2c). All enzymes differ significantly in the size of the substrate binding pocket (fdeE > RgF8H > LjF8H), as shown in Supplementary Fig. S1, whereas the hydrophobic residues mostly overlap, and some are even identical. The polar or aromatic residues differentiate significantly, which may be responsible for variation in substrate specificity. Both fdeE and LjF8H share the same electrostatic surfaces with similar residues distributed in space, while in RgF8H, basic residues R224 and K369 are oriented towards the substrate, changing the surface potential, which should be responsible for different binding affinities and hence, different substrate specificities. Docking of naringenin and quercetin to RgF8H and fdeE results in almost all binding positions overlapping near the FAD with the correct orientation for C8 hydroxylation, with a few low-score results where the B ring faces FAD. This is interesting to note, as no reaction towards C3’ or C4’ (chrysin/pinocembrin) was observed in the case of RgF8H, and also no activity towards quercetin or other flavonols. Additionally, only in the case of fdeE isoflavone genistein might be docked in the correct orientation for hydroxylation, which was further confirmed experimentally (Supplementary Table S4).

### Confirmation of predicted activity

**Figure 3:**
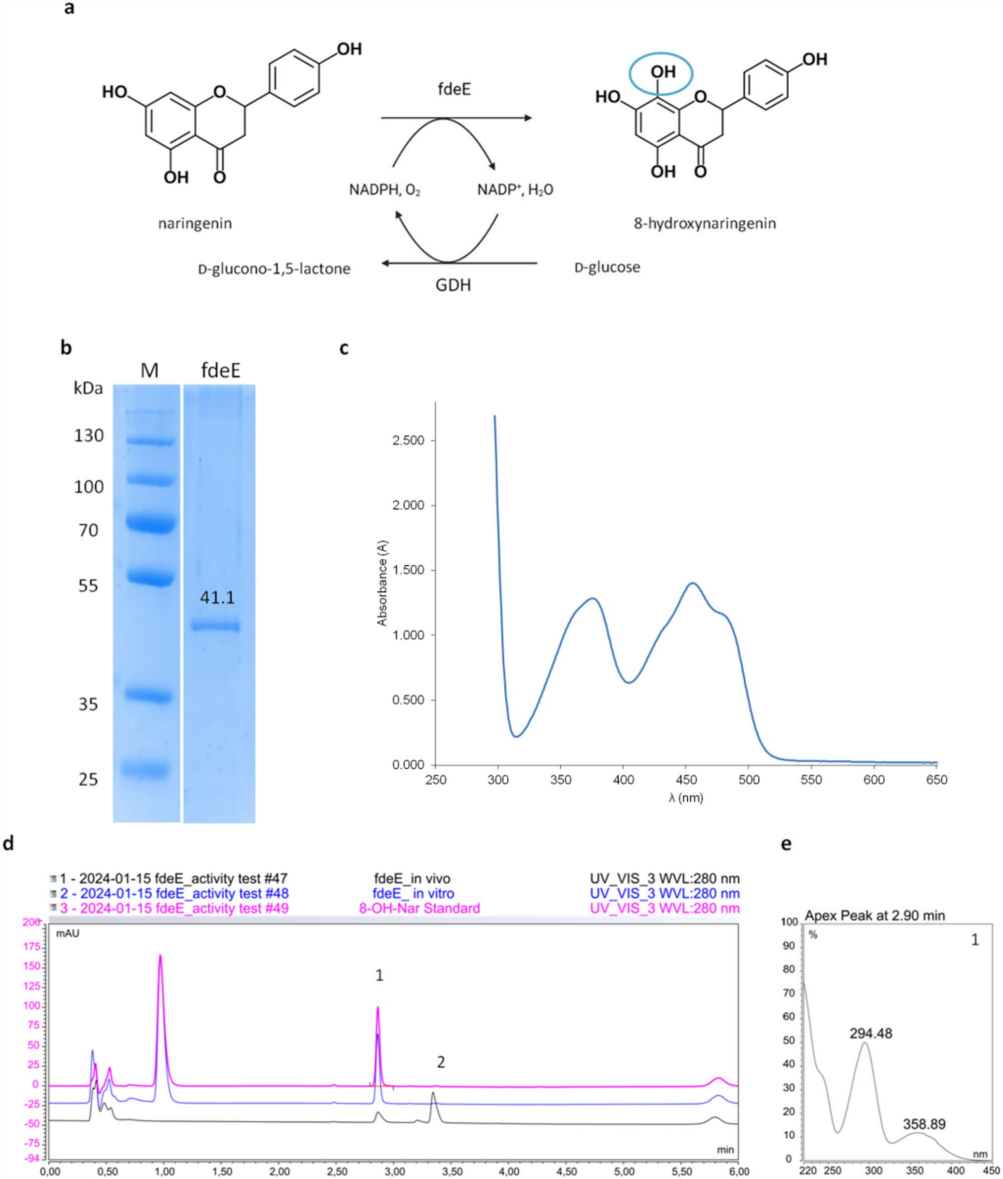
(**a**) Scheme of the hydroxylation reaction of naringenin by fdeE. (**b**) SDS-PAGE gel electrophoresis of recombinant hydroxylases: marker (line 1), purified (2 × HisTrap) fraction of fdeE (line 2, full SDS-Page electrophoresis gel picture can be found in Supplementary material), (**c**) UV-Vis spectrum of purified fdeE. (**d**) The UPLC analysis of reaction products catalyzed by the recombinant protein in vivo and in vitro reaction (pink line—standard of 8-hydroxynaringenin, blue line—in vitro reaction, black line—in vivo reaction). The UPLC profiles were monitored by a photodiode array detector. 1: 8-hydroxynaringenin, 2: naringenin. (**e**) UV–Vis spectrum of 8-hydroxynaringenin.

The enzyme was produced using transformed *E. coli* cells. A codon-optimized sequence of fdeE was ordered in pSEVA182 vector with specific restriction sites for Golden Standard MoClo assembly. Then, the two sets of vectors were prepared—for inducible expression with RhaS/pRhaBAD promoter and with pJ23100 strong constitutive synthetic promoter (Supplementary Table S2). Overexpression of protein upon induction with rhamnose in *E. coli* BL21 (DE3) cells and subsequent purification by IMAC Ni^2+^ affinity chromatography enabled in vitro reactions and protein analysis via SDS-PAGE. Based on SDS-PAGE analysis (for qualitative purity of purified protein), Bradford analysis and further calculation, we have shown that the production and purification of the enzyme is efficient (Fig. 3b, Supplementary S2) and of approximate levels of pure protein produced are > 100 mg/L of culture. Characteristic of FMO, the yellow color was observed for the purified enzyme fraction solution, what was confirmed with the the UV–Vis spectrium of purified fdeE with absorption spectra characteristic for FAD absorbance and FMOs (Fig. 3c).

A constitutive vector was used for in vivo activity, while a rhamnose-induced vector with an additional cofactor regeneration system was used for the in vitro reaction (Fig. 3a). Analysis of the products obtained by UPLC-DAD revealed the presence of a peak overlapping with the retention time and UV spectrum of the 8-hydroxynaringenin standard, both in case of in vivo and in vitro reactions (Fig. 3d,e).

### Enzyme characterization

Investigated flavonoids hydroxylase are flavin-containing enzymes, therefore an important parameter to evaluate is the uncoupling rate of the peroxyflavin intermediate formed upon reaction with molecular oxygen^43^. The uncoupling rate can be determined by measurement of coenzyme (NADPH or NADH) oxidation, after the initial saturation of the reaction buffer with oxygen^44^. In the case of purified fdeE over 30 min of incubation, the NADPH concentration did not change (Fig. S3a) indicating almost no uncoupling. For NADH, we observed a slight decrease in concentration (Fig. S3b), which may be due to trace contamination of other enzymes that we were unable to remove as well as detect. More importantly, the concentration of each coenzyme decreased when naringenin was added to the reaction mixture (Fig. S3), although the results clearly show that NADPH is more preferred by the described enzyme than NADH, nevertheless, both coenzymes were accepted and we observed product formation for both. However, due to the preferential use of NADPH by fdeE in the subsequent assays we performed to characterize the properties of the enzyme, we already used only NADPH as the hydride donor. Interestingly, the incubated enzyme retained the yellow color in solution over the incubation time (no change also in UV–Vis absorbance profile of the enzyme solution was observed upon addition of NADPH), which might be linked to stable hydroperoxyflavin, thus the superoxidized form of enzyme, although no characteristic absorption spectra for this intermediate was detected (Fig. S4)^45–47^. Hydrogen peroxide produced as a result of decupling might damage the cell and lead to unwanted oxidation of both substrate and/or product in vitro and in vivo reactions.

Little is known about these FMOs, so we analyzed other biochemical characteristics of the enzyme to find out, whether sequence identity to RgF8H would result in similar substrate scope and optimal biochemical parameters. The results of these studies are presented in Table 1a and Supplementary Fig. S5. fdeE shows best activity in the range 20–30 °C, above > 60% (Fig. S5a). At temperatures above 40–45 °C, only negligible activity was observed, and protein aggregates were easily visible. The enzyme was most active in an acidic environment, with the optimum pH for the reaction equal to 5.0 (Fig S5b). However, under these conditions, the enzyme is rapidly denatured. The ThermoFAD assay showed that apparent melting temperature (Tmapp) in the buffer of pH ≤ 5.5 in the temperature range of 40–45 °C (Fig. S5f.). The use of a more alkaline environment significantly increased the stability of the enzyme. In buffer pH 6–6.5, denaturation of the enzyme was observed at 54 °C. The enzyme was most stable at pH 7–7.5 (denaturation at 65–70 °C) and pH 8–8.5 (62–69 °C), while at pH 9–10 it melts at about 58 °C. Furthermore, it was observed that the type of buffer also affected the stability of the protein (pH = 8, phosphate buffer—66 °C, Tris–HCl—69 °C buffer). Therefore, a phosphate buffer of pH 7.5 was used for the enzyme in further studies, ensuring both high stability and activity. The most interesting finding of this test is the sensitivity to buffer concentration and ionic strength. The best results were obtained with a 10–15 mM buffer, a slightly worse result was observed in a 25 mM buffer, while the use of 50–100 mM buffers was associated with a decrease in enzymatic activity by half (Fig S5d). Concerning ionic strength, a decrease in the relative activity below 70% was observed at salt concentrations of 0.3 M (Fig. S5e). The addition of a small amount of the co-solvent DMSO had a beneficial effect on the conversion (> 5%), while the addition of a larger amount of either solvent was associated with a decrease in enzyme activity (Fig. S5c). At temperatures above 40 °C, the enzyme lost its activity in less than one hour (Fig. S5g), while holding it at temperatures up to 40°C for three hours did not affect it. Due to the extreme instability of the enzyme during storage, complete inactivation was observed after 1 day at − 20 °C. The addition of glycerol maintained enzyme activity longer but did not stop enzyme inactivation. Furthermore, replacing the buffer after purification and before the storage also had a positive effect (Supplementary Fig. S6). For the purified fraction, the best storage buffer is 25 mM Tris–HCl pH 8.0 with the addition of 10% v/v glycerol. However, storage at 4 °C resulted in a loss of activity in less than one day after purification, and the addition of BSA (0.1–1.0 mg range) and/or FAD (10–1000 µM) did not affect stability despite the addition of glycerol. For this reason, some subsequent steps for this enzyme were carried out using crude protein extracts that retained enzyme stability > 80% after 30 days of storage at − 20 °C.

### Determination of reaction product structures

The in vitro reaction was performed with the addition of a reducing agent (DTT) and internal standard (dibenzofuran), to determine substrate acceptance towards flavonoids and phenolic compounds. The results were assessed by UPLC-DAD and LC–MS analysis. The substrate library containing 49 compounds is shown in Supplementary Table S3. Regarding flavonoids, the enzyme showed broader substrate specificity than the RgF8H described earlier^25^ (Table 1b). fdeE was active towards 17 of tested substrates and hydroxylated flavanones and flavones, similar to the RgF8H, and also show activity towards isoflavones and flavonols. The location of the hydroxyl groups in the A ring of the substrate was noteworthy. A correlation between the enzyme activity and the presence of hydroxyl groups at C-5 and C-7 carbon atoms of the substrate was found. Each reaction product was characterized by UV–Vis absorbance maxima and by electrospray ionization mass spectroscopy (ESI/MS) (Table 1b and Supplementary Fig. S7), and detailed descriptions of the LC/MS analysis can be found in the Supplementary Files, section—Products identification. The similarity of the investigated bacterial hydroxylase to the eukaryotic enzyme RgF8H ends with activity towards flavones and flavanones, as fdeE has broader substrate scope. These results are complemented by the sequence homology of the two enzymes (53%) shown by in silico amino acid sequence analysis and also in 3D models putting the two enzymes on an equal footing in terms of mechanism of action rather than substrate acceptance (Fig. 2c). Interestingly, for the tested substrates, we observed enzymatic activity of both of them only against flavonoids, confirming the assumption of a specific flavonoid degradation pathway already described in *H. seropedicae* SmR1^26^ and probably occurring in *R. glutinis* KCh735.

### The formation rate of hydroxyflavonoids

Due to the low stability of the hydroxylation products, studies of product formation and degradation over time were carried out. Five substrates were chosen for the in vivo study: naringenin, luteolin, apigenin, quercetin, and chrysin. We decided to limit the range of substrates tested and select those whose stabilized production may be of interest to the industry due to their already proven properties, yet very low stability.

**Figure 4:**
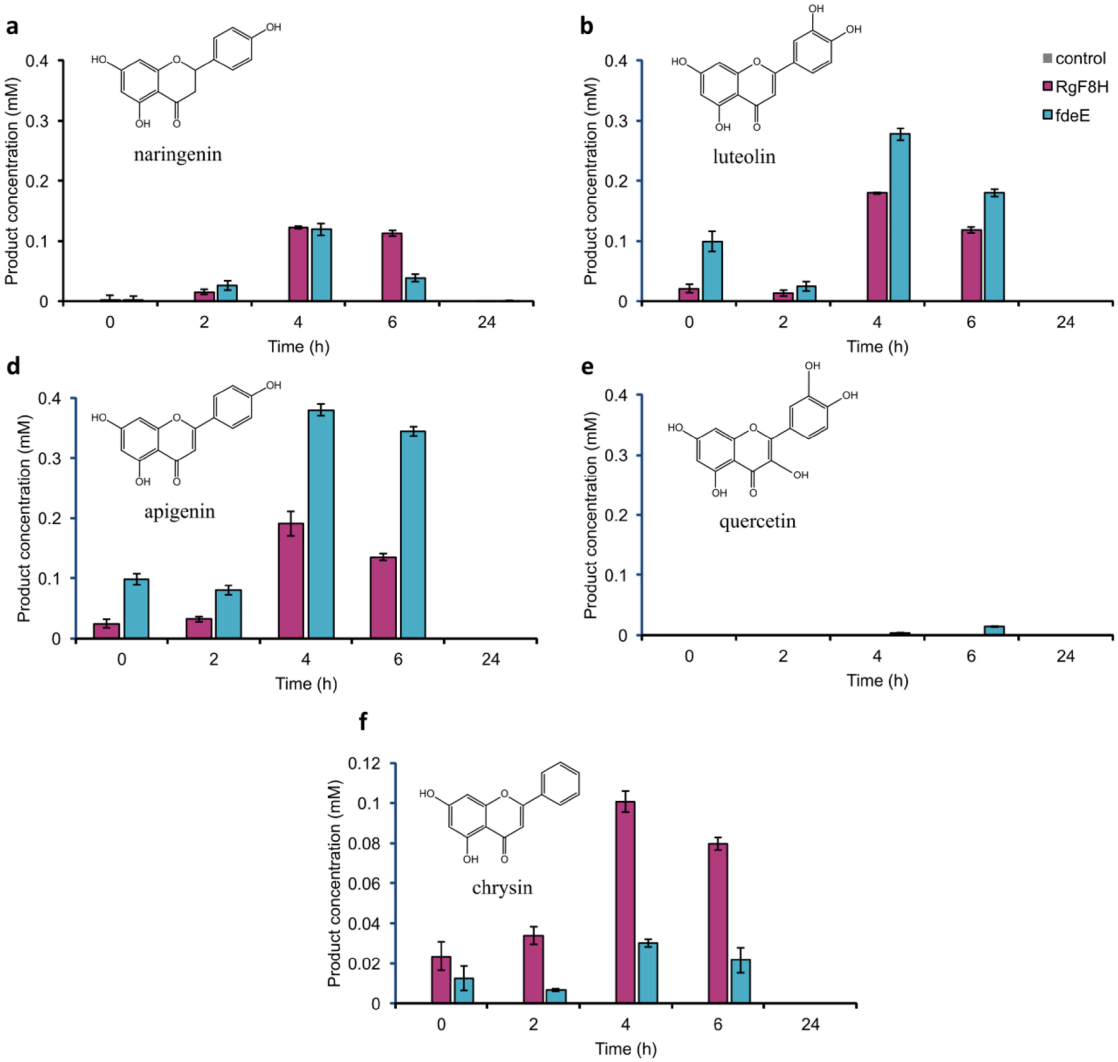
Degradation of C-8 hydroxylation products over time in vivo at an initial substrate concentration of 1 mM: (**a**) naringenin, (**b**) luteolin, (**c**) apigenin, (**d**) quercetin, (**e**) chrysin.

Flavonoids that differ in their hydroxylation ratio and hence susceptibility for further over-oxidation in the reaction mixture. Figure 4 demonstrates that all hydroxylation products were degraded after 24 h of cultivation. In addition, we also used RgF8H in our study to compare the activity of all F8-FMOs already identified and described in the available literature. The highest activity was observed for fdeE, which performed the hydroxylation reaction rapidly, and also the product was degraded within 6–8 h. Degradation of the product or substrate was closely related to the color change of the medium. Control cultures with naringenin, apigenin, and chrysin did not change color (Supplementary Fig. S8), while visible color changes were observed in control cultures with quercetin and luteolin, indicating that these substrates were degraded even by untransformed *E. coli* DH5α cells during the 24-h cultivation which is also confirmed by the UPLC results (Supplementary Figs. S9, 10). In the case of quercetin, a dark brown color was observed as early as 6 h in fdeE cultures.

**Figure 5:**
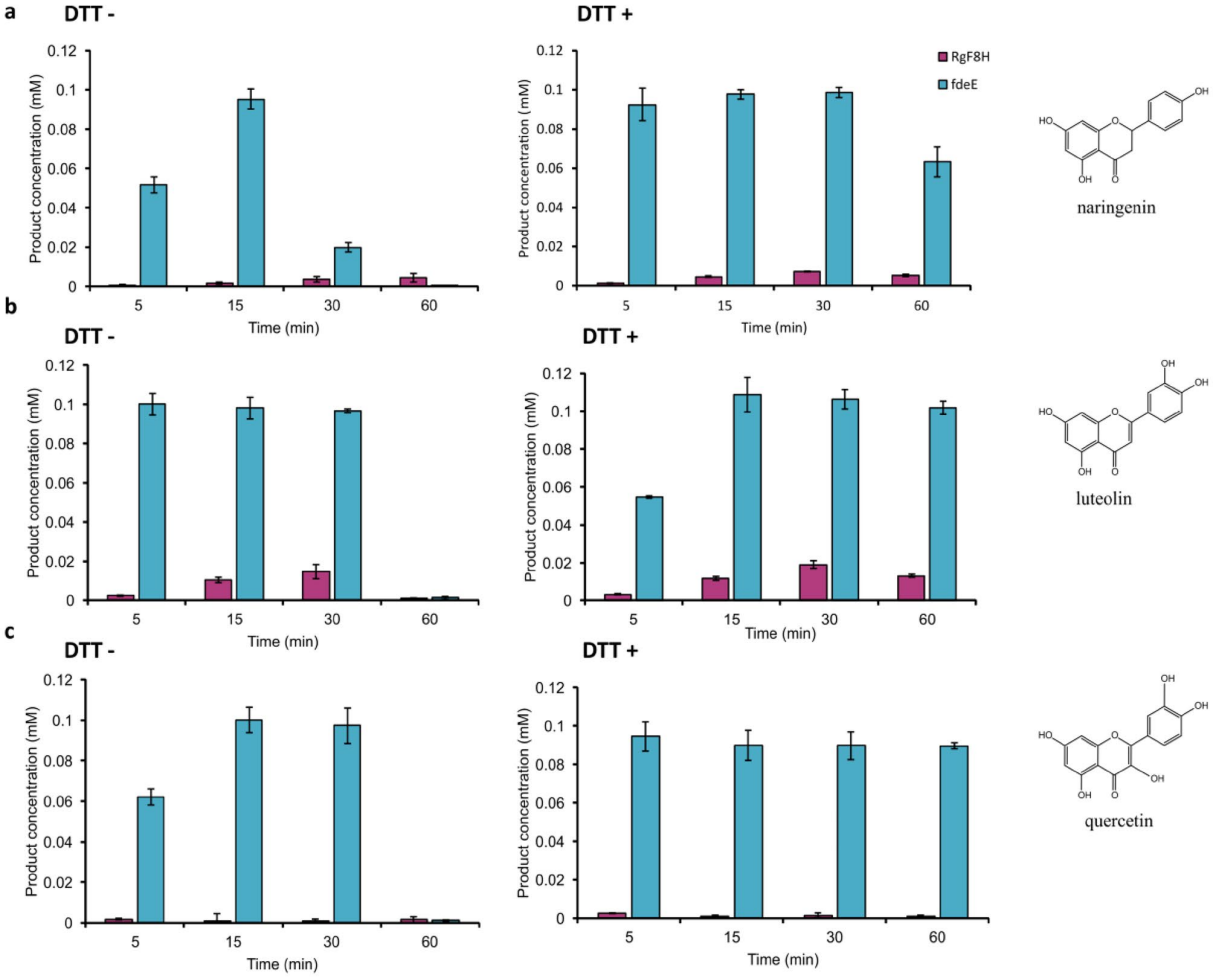
Stability of C-8 hydroxylation products in vitro reaction over time at an initial substrate concentration of 0.1 mM: (**a**) naringenin, (**b**) luteolin, (**c**) quercetin, without and with 25 mM DTT addition.

Studies on C-8 hydroxy derivatives of flavonoids have shown that hydroxylated products exhibit enhanced antioxidant properties^48^. This activity also has implications for preparative reactions, as the resulting products are susceptible to oxygen-induced degradation. Therefore, the oxygen required for the reaction may also promote product degradation. To test, whether lowering the redox potential of the entire reaction mixture, could affect product degradation without significantly affecting the progress of the reaction, we experimented with the mild addition of a reducing agent to an in vitro reaction with naringenin and quercetin as substrates. The best results were obtained using 5–25 mM dithiothreitol (DTT), followed by 5 mM thiosulphate, but with already half the reaction yield (Supplementary Fig. S11), similar results were obtained for sodium dithionite. In contrast, the addition of cysteine did not affect the stability of the reaction products. On the basis of the tests performed with the addition of a reducing agent to the in vitro reaction, we decided to investigate the stability and yield of the products using 25 mM DTT in the reaction mixture. The two enzymes fdeE and RgF8H were used, and naringenin, luteolin and, quercetin were tested as substrates. The highest conversions were observed for the fdeE enzyme for each of the three tested substrates, while reaction efficiencies of an order of magnitude lower were observed for RgF8H. For comparison, trials without the addition of a reducing agent were carried out, for which almost all products were degraded within 30 min (Fig. 5 DTT-). In the case of naringenin, half of the product was oxidized after 15 min with each of the enzymes used. fdeE converted > 99% of the luteolin and quercetin within 15 min but products were also not detected if the reaction lasted longer than 30 min, indicating their degradation.

For test with and without the addition of DTT, for which we obtained the best results in our preliminary test (Fig. S11), we chose three compounds from our previous test, naringenin, luteolin, and quercetin. These substrates belong to different subclasses of flavonoids (flavanones, flavones, and flavanols), differ in the hydroxylation ratio, and among previously tested were the most prone for undesired degradation, which allowed us to verify the potential stability of formed products. A protective effect for 8-hydroxynaringenin was evident for the first 30 min of the reaction, running the reaction longer allowed half of the products to be retained for each of the enzymes tested (Fig. 5a). Different results were obtained for luteolin, where lowering the redox potential of the reaction environment resulted in stable yields as well as product retention. For fdeE, we observed > 99% conversion already after 15 min and no change in product concentration for a further 45 min. RgF8H was also active towards luteolin, but with a lower yield, and also for this enzyme, we were able to observe the reaction product after 60 min (Fig. 5b). During the in vivo and in vitro reaction, the hydroxylation product of quercetin at the C-8 position was degraded almost immediately. Application of DTT allowed the product to be retained even after 60 min of reaction. The bacterial enzyme—fdeE, showed activity towards this substrate, converting > 99% of quercetin within 5 min, and due to the lowering of the redox potential of the reaction medium, the product remained stable for another 60 min (Fig. 5c). We also observed that the addition of DTT to the reaction mixture affected both product stability and reaction rate. Lowering the redox potential in the reaction with naringenin and quercetin decreased the rate of product degradation, especially in the first 5 min of the reaction (Fig. 5a,c). A completely opposite relationship was shown for luteolin, for which the addition of DTT stabilized the hydroxyproduct, but also caused it to form almost half as fast in the first 5 min (Fig. 5b). This clearly shows that, when choosing a reducing agent, we need to be careful and thoroughly check their effect on the reaction process with individual substrates as both effects, lowering the product degradation and reaction rate can be observed.

Additionally, an attempt to stabilize the products by methylation is not possible due to the demethylating activity of the hydroxylases. This was confirmed in the case of wogonin, which contains a methyl group at the C-8 carbon atom that was oxidized—leading to demethylation and conversion of wogonin to 8-hydroxychrysin (Supplementary Fig. S12). UPLC-DAD analysis of the reaction—retention time and UV–Vis spectrum indicated clearly the hydroxylation product of chrysin and the demethylation of wogonin.

### Kinetic parameters

Preliminary tests showed that the UV spectra of the individual flavonoid compounds and the coenzyme, whose consumption we initially planned to use to determine the kinetic parameters of the enzyme, overlapped. Making spectrophotometric measurements of the time course of the reaction impossible. Being aware that it would be impractical to determine the kinetic parameters from the NADPH concentration, we decided to use parameters derived directly from the product formation progress curve. This approach does not require a vast amount of samples to be made and gives the possibility to estimate V_max_ and K_m_ from a single reaction^49^. We obtained the highest values of the kinetic parameters for luteolin and chrysin, while quercetin was the slowest transformed substrate (Table 2, Supplementary Fig. S13), which is in agreement with our previously obtained results. Due to the low stability of the compounds obtained and despite the use of the reducing agent in the reaction medium, it should be noted that the results achieved may be subject to errors, caused by the inevitable partial decomposition of the product. However, a clear conclusion of evolutionary adaptation of fdeE to degrade naringenin, the most common flavonoid in plants is clearly observed.

**Table 2:** Calculated kinetic parameters for five substrates, their initial reaction concentrations 0.05mM.

Substrate	V_max_ (mM/s)	K_m_ (mM)	k_cat_ (s^−1^)	k_cat_/K_m_ (mM^−1^ s^−1^)
Naringenin	3.49 × 10^−5^	4.22 × 10^−4^	2.77 × 10^−2^	65.54
Apigenin	6.70 × 10^−5^	2.02 × 10^−2^	5.32 × 10^−2^	2.63
Luteolin	1.02 × 10^−4^	5.18 × 10^−2^	8.11 × 10^−2^	1.57
Quercetin	8.54 × 10^−6^	1.49 × 10^−2^	6.78 × 10^−3^	0.45
Chrysin	1.47 × 10^−3^	4.31 × 10^−1^	1.17	2.72

### 8-Hydroxyquercetin stabilization, purification and identification

In our previous studies, we obtained hydroxy derivatives of flavones and flavanones^25^, however, the characterization of fdeE enabled also a route to hydroxylated flavonols and isoflavones (Table 1b).

Because of the many challenges involved in the preparation of these extremely valuable compounds containing so many hydroxyl groups (like photo-lability, insolubility in water, or arduous purification), we set out to develop a stable and efficient way to produce and purify them^50–52^. Quercetin was used as a substrate for this study, firstly because of its antioxidant properties, but more importantly because of the range of problems to be solved for its rapid oxidation that should occur during extraction and purification. Being aware of the problem of extracting the quercetin product, we tested three different approaches. The preparative conversion of quercetin after 24 h of reaction with fdeE ended at conversion of 17%, which was equivalent to 17.1 mg of 8-hydroxyquercetin obtained (Supplementary Table S5). Assuming low losses during purification, the amount of product obtained after purification should be about 5 mg, due to the division of the reaction mixture into 3 parts. Such results were obtained only for the sample extracted with ethyl acetate, 5.16 mg, thus, the purification efficiency was more than 90%. The use of 1-butanol resulted in a loss of more than half of the product. Lyophilization of the entire mixture proved to be the worst method of product purification, as degradation of the compounds during the chromatography was evident after already a dissolution and filtration. This was confirmed by the negligible mass of the final product obtained.

**Figure 6:**
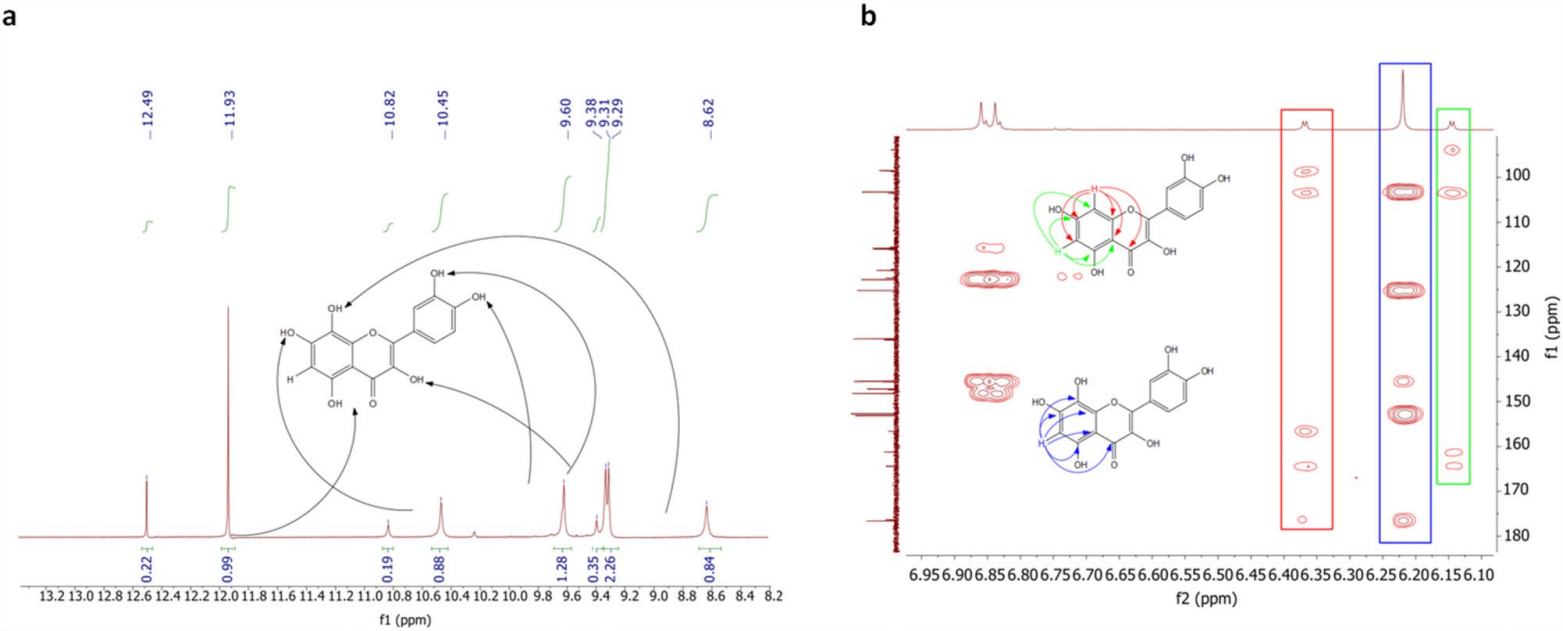
(**a**) ^1^H-NMR (600 MHz, DMSO-*d*_*6*_) spectrum part of 8-hydroxyquercetin (major) and quercetin (minor) mixture—signals from hydroxyl groups. (**b**) ^1^H-^13^C NMR (HMBC) (600 MHz, DMSO-*d*_*6*_) spectrum of 8-hydroxyquercetin (major) and quercetin (minor) mixture with characteristic signals indicating specific site of hydroxylation.

The structure of the product was confirmed by NMR spectrometry, which unambiguously showed the introduction of the hydroxyl group to the C-8 carbon and evident peak shifts as the quercetin was added in 20% (mass/mass) to the analysis (Fig. 6 and Supplementary File: Figs. S14–S17). Detailed descriptions of the NMR results can be found in the Supplementary Files, under—Products identification.

## Discussion

The search for new enzymes capable of regioselective transfer of oxygen to aromatic compounds is an important branch of current science^53^. Screening tests popularly performed to find microbial organisms with interesting catalytic properties are now in decline^54^. The strength of the new strategy based on in silico screening creates the possibility of discovering new enzymes very quickly or extending the knowledge of already partially described biocatalysts^55,56^. In contrast to the earlier approach, there is no need to set up huge numbers (preliminary tests) of samples on such a massive scale and verify them one by one in the hope of finding a strain catalyzing whatever type of reaction. A thorough analysis of the structure of the protein sought and a search for homology with protein sequences already available in databases is sufficient^57^. Reflecting on the core of the described transformations, i.e. characterization of the metabolic pathway or enzyme, is, according to the authors of the paper, the greatest advantage of the strategy described. Even if the structural information relates to enzymes from evolutionarily distant organisms, there is a fairly high probability of homologous action. Moreover, their mechanism of action may be the same, but the range of catalytic possibilities is much more interesting.

The described in this paper characterization of FMO is an ideal example of expansion of dormant catalytic potential that lies hidden within various degradation pathways, that might find application in biocatalysis and synthetic biology. Continued work on this enzyme has greatly expanded the library of products obtained using C-8 hydroxylases to include flavonols and isoflavones, in comparison to the already described enzyme, which is highly specific for flavones and flavanones only^25^.

In the example presented in this paper, we have drawn on recent work^25,48,58^ and exploited the unique presence, as well as absence, of characteristic motifs and structural similarity allowing us to identify a broad spectrum of fdeE activity. We then carried out a phylogenetic analysis, which showed that the sequences of the used enzymes (fdeE and RgF8H) stand out and form a new branch of enzymes that is much closer to uncharacterized monooxygenases than CYP, DOX, plant-FMO, or Rieske enzymes. Furthermore, among the uncharacterized monooxygenases the LjF8H^33^ sequence, described as a plant C-8 hydroxylase is lacking the F motif in its structure, which is conserved among all known plant-derived FMOs. These results suggest that fdeE^27^ and RgF8H^25^, may belong to a larger group of similar FMOs including also plant-derived enzymes, however, these were not sufficiently described in the literature, i.e. LjF8H^33^. We have tried also to express LjF8H in *E. coli* cells unfortunately, similar to studies of Hiraga and co-authors^33^, our efforts were unsuccessful (data not shown). This, therefore, confirmed that *E. coli* is not a suitable host for the production of this enzyme.

However, fdeE, shares most biochemical parameters with other FAD-dependent enzymes described in the literature. Like LjF8H^33^ and RgF8H^25^, it showed an absolute requirement for NADPH, while the addition of FAD to the reaction mixture containing the NADPH coenzyme did not result in changes in activity, as also was described for LjF8H^33^. This correlates with the hypothesis that FAD may remain tightly bound to these enzymes^59^. This is in contrast to RgF8H, which activity increased markedly when FAD was added to the reaction mixture, what might indicate that additional loop observed in the structure of the enzyme may have other role than aid of FAD binding^25^.

For fdeE from *H. seropedicae* SmR1, activity against naringenin (flavanone) has been described^26,27^ and there are no reports of attempts to use this enzyme against other flavonoids. In our work, we have considerably expanded the scope of its possibilities. In addition, the use of a cofactor regeneration system involving the GDH enzyme from *B. megaterium*^60^ and the addition of DTT allowed the reaction to be carried out on a semi-preparative scale, which, to the best of our knowledge, has not been described before.

Kinetic analysis of the fdeE allows to compare its activity with other known flavonoid hydroxylases, which also share *ortho-*hydroxylation reaction pattern. The kinetic parameters of enzymes exhibiting similar enzymatic activity collected from the literature are summarized in Table 3. Although reports on kinetic values are rather scarce, presented in different units requiring recalculation and mostly reported for compounds other than tested, we can conclude that among FMOs fdeE exhibits a much higher turnover number comparable to P450 monooxygenases. Thus, due to its higher substrate affinity, relatively high protein yield and no requirements for specific reductase makes fdeE a more attractive candidate for preparative or industrial applications.

**Table 3:** Collected literature kinetic parameters for determined enzymes catalyzing similar reactions.

Enzyme	Type of enzyme	Substrate	K_m_	k_cat_	V _max_	Source
ObF8H-1	C-8 Rieske	Salvigenin	1.6 µM	–	60.3 pkat/mg	^61^
CsFMO	FMO	Quercetin	9 µM	–	133 µkat/kg	^62^
		Luteolin	10 µM	–	72 µkat/kg	^62^
HpaB	FMO	Naringenin	192 µM	0.1 s^−1^	–	^65^
ObF6H	P450	Genkwanin	0.2 µM	4.97 s^−1^	–	^63^
		Naringenin-7-methyl	1.29 µM	3.25 s^−1^	–	^63^
		Apigenin-7,4′-dimetyl	0.15 µM	1.64 s^−1^	–	^63^
AaeAPO	P450	Apigenin	290 µM	26 s^−1^	–	^64^
BM3_M13	P450	Naringenin	446 µM	1.955 s^−1^	–	^66^
MtFBH-4	P450	Apigenin	2.58 µM	–	1.15 µM/h	^66^
MtFBH-5	P450	Apigenin	24.64 µM	–	1.44 µM/h	^66^
AtF3’H	P450	Naringenin	1.2 µM	2.1 µmol/s*µg	137.4 µM/s	^67^
FvF3’H	P450	Naringenin	1.0 µM	10.6 µmol/s*µg	82.7 µM/s	^67^
TeF3’H	P450	Naringenin	3.0 µM	17.0 µmol/s*µg	38.7 µM/s	^67^

Our work focuses on quercetin, the stabilization of which is of interest to many research groups. It exhibits photostability in propylene glycol solutions^50^, while its photodegradation was observed in creams unless protected in lipid micelles^68^. In addition, degradation of quercetin in the dark dissolved in an alkaline medium^51^, treatment with radical generators, or electrochemical oxidation in ethanol^52^ was observed. In nucleophilic solvents, there is an addition of the solvent molecule to the 2,3 double bond of quercetin and oxidation by air oxygen. The same mechanism operated when quercetin was dissolved in alkaline solutions. The researchers showed that hydroxyl groups at the 3, 3′, and 4′ positions are involved not only in the antioxidant activity of quercetin but also in its photostability^69^. Attempts to stabilize the quercetin molecule have been made, including forming a complex with iron and aluminium^70^. Due to all the factors mentioned above, obtaining pure quercetin derivatives without disturbing their structure is difficult to achieve. Even more so in light of their dynamic oxidation by lyophilization^69^, which is also confirmed by the results of our study.

The efficacy of reducing agents such as ascorbic acid, dithiothreitol, and tris(2-carboxyethyl)phosphite for highly unstable catechins has already been described. Among the compounds used, the best results were obtained for samples with the addition of DTT^71^. In our work, the maneuver with the manipulation of the reaction environment proved to be an ideal way not only to stabilize the substrate but also to efficiently carry out the hydroxylation reaction, proceeding without interference for up to 24 h and yielding purified 8-hydroxyquercetin at a concentration of 0.16 g/L.

### Supplementary Information

Supplementary Information.

## Data Availability

All data generated or analyzed during this study are included in this published article and its supplementary information files. All plasmids, genes or samples of products are available upon request.
