# Supplementary material for: Novel enzymatic route to the synthesis of C-8 hydroxyflavonoids including flavonols and isoflavones
Source: Sci Rep. 2024 Aug 6;14:18217. doi: 10.1038/s41598-024-68513-5 (PMC11303751; doi:10.1038/s41598-024-68513-5)
Supplement: Supplementary file 1 — Supplementary Information. [file 41598_2024_68513_MOESM1_ESM.docx]

Supplementary materials

Novel Enzymatic Route to the Synthesis of C-8 Hydroxyflavonoids Including Flavonols and Isoflavones..

Kinga Dulak^a^, Sandra Sordon^a^, Agata Matera ^a^, Aleksandra Wilczak^b^, Ewa Huszcza^a^, Jarosław Popłoński*^a^

^a^Department of Food Chemistry and Biocatalysis, Wroclaw University of Environmental and Life Sciences, Wroclaw, Poland ([agata.matera@upwr.edu.pl](mailto:agata.matera@upwr.edu.pl)), ([sandra.sordon@upwr.edu.pl](mailto:sandra.sordon@upwr.edu.pl)), (ewa.huszcza@upwr.edu.pl)

^b^Hirszfeld Institute of Immunology and Experimental Therapy, Polish Academy of Sciences, Wroclaw, Poland (aleksadnra.wilczak@gmail.com)

List of content

[Supplementary Figure S1. Predicted 3D models 2](#_Toc163851043)

[Supplementary Figure S2. SDS-PAGE gel electrophoresis of recombinant hydroxylases 2](#_Toc163851044)

[Supplementary Figure S3. Oxidation of cosubstrate 2](#_Toc163851045)

[Supplementary Figure S4. UV-Vis absorbance spectra 3](#_Toc163851046)

[Supplementary Figure S5. *In vitro* analysis of the biochemical properties 3](#_Toc163851047)

[Supplementary Figure S6. Relative enzymatic activity measured for NADPH consumption in reaction 4](#_Toc163851048)

[Supplementary Figure S7. LC-MS analysis of hydroxyl products. 9](#_Toc163851049)

[Supplementary Figure S8. Degradation of C-8 hydroxylation products over time *in vivo* reaction – culture colour after 24 hours. 9](#_Toc163851050)

[Supplementary Figure S9. Level of degradation of substrates over time *in vivo* reaction. 9](#_Toc163851051)

[Supplementary Figure S10. Formation of C-8 hydroxylation products over time *in vivo* reaction 10](#_Toc163851052)

[Supplementary Figure S11. Effect of reducing agents on the stability of hydroxy derivatives 11](#_Toc163851053)

[Supplementary Figure S12. (a) Scheme of the chrysin hydroxylation reaction. (b) Scheme of the wogonin demethylation reaction. (c) UPLC-DAD analysis. (d) UV-Vis absorption spectrum. 11](#_Toc163851054)

[Supplementary Figure S13. Formation curve of 8-hydroxy derivatives. 12](#_Toc163851055)

[Supplementary Figure S14. ^1^H-NMR (600 MHz, DMSO-*d_6_*) spectrum of 8-hydroxyquercetin. 12](#_Toc163851056)

[Supplementary Figure S15. ^13^C-NMR (600 MHz, DMSO-*d_6_*) spectrum of 8-hydroxyquercetin. 13](#_Toc163851057)

[Supplementary Figure S16. ^1^H-^13^C NMR (HSQC) (600 MHz, DMSO-*d_6_*) spectrum of 8-hydroxyquercetin. 13](#_Toc163851058)

[Supplementary Figure S17. ^1^H-^13^C NMR (HMBC) (600 MHz, DMSO-*d_6_*) spectrum of 8-hydroxyquercetin. 14](#_Toc163851059)

[Supplementary Table S1. Calculated parameters of substrate binding pocket volume for the predicted 3D models 14](#_Toc163851060)

[Supplementary Table S2. Accession numbers and origin of amino acid sequences used in the phylogenetic analysis. 14](#_Toc163851061)

[Supplementary Table S3. Primer sequences and description of transcription units. 15](#_Toc163851062)

[Supplementary Table S4. Structures of substrates used in this work. 16](#_Toc163851063)

[Supplementary Table S5. Reaction yield and purification efficiencies. 18](#_Toc163851064)

[Products identification 19](#_Toc163851065)


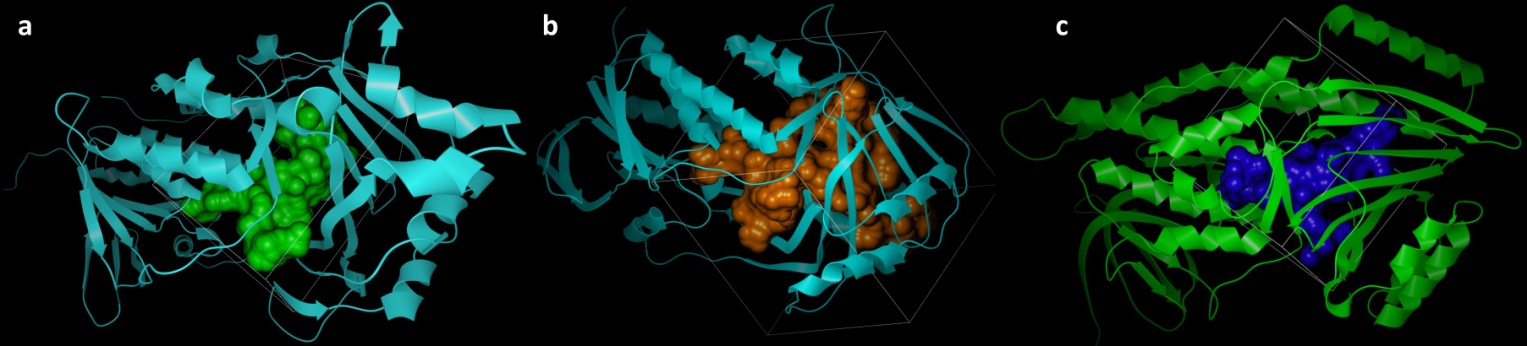


# Supplementary Figure S1. Predicted 3D models of (a) – RgF8H, (b) – fdeE and (c) – LjF8H constructed with Caver Analyst 2.0 software^1^ with cavities detected using default software cavity calculation (Probe 1.4, Large probe 3.0).


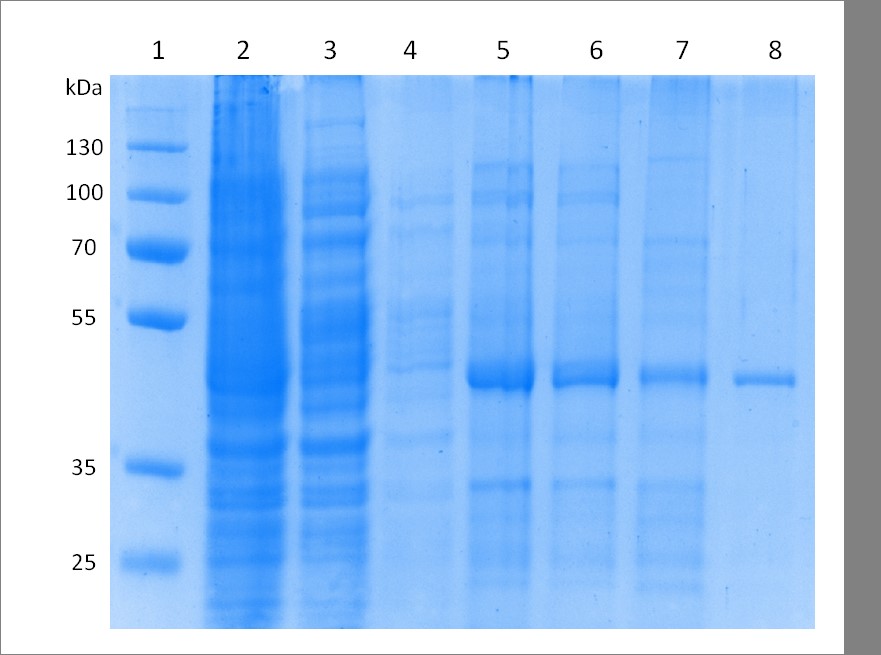


# Supplementary Figure S2. SDS-PAGE gel electrophoresis of recombinant hydroxylases: marker (line 1), crude (line 2), filtrate (line 3), wash (line4) collected fraction of enzyme (line 5-7), purified 2x HisTrap enzyme fraction (line 8). Expected mass of purified fdeE fraction: 41.1 kDa.


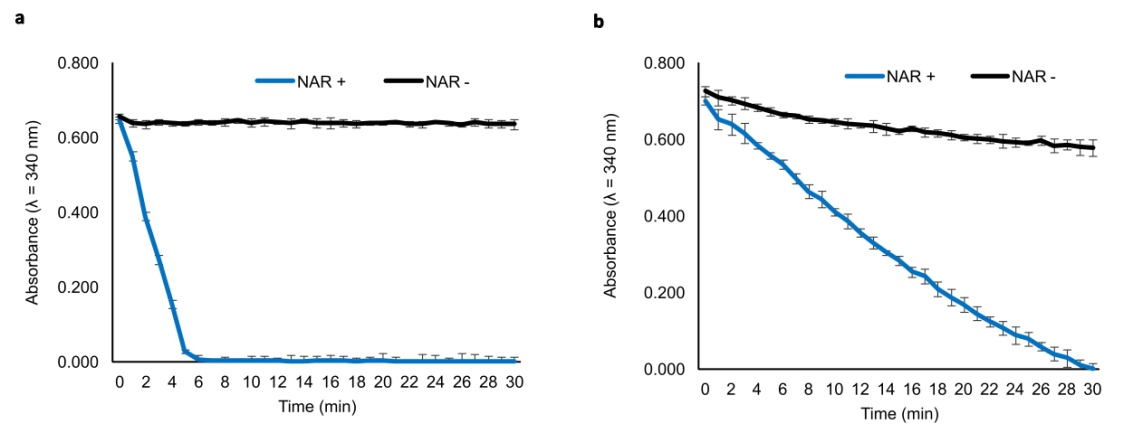


# Supplementary Figure S3. Oxidation of cosubstrate (a) NADPH and (b) NADH by fdeE with and without naringenin addition in an *in vitro* reaction. The initial cosubstrate concentration was 1 mM.


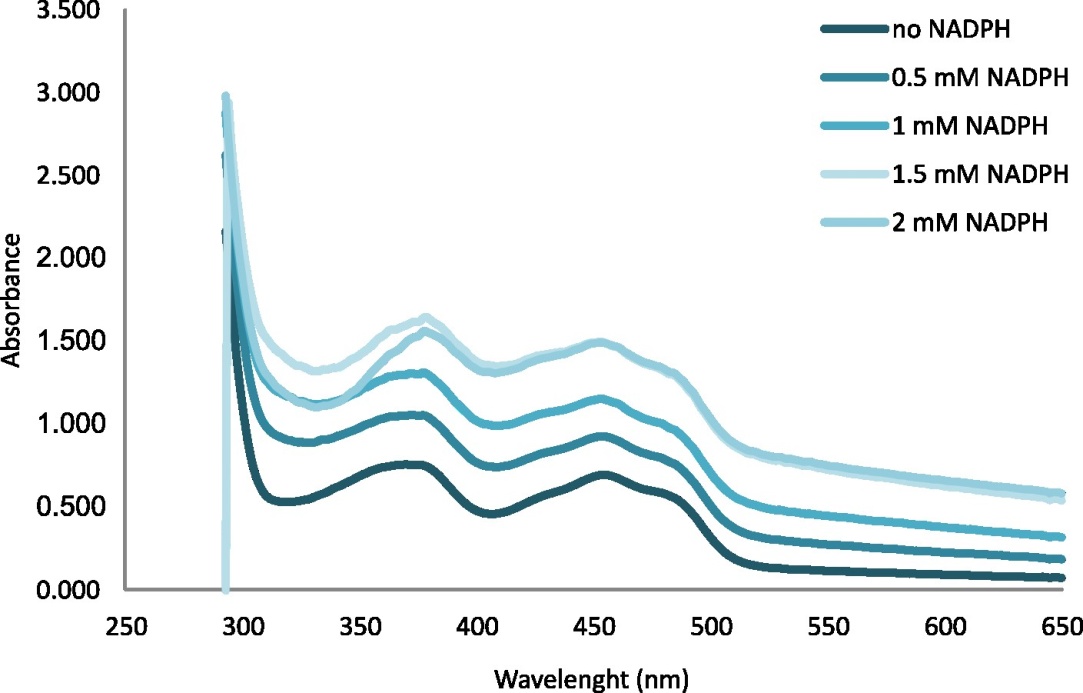


# Supplementary Figure S4. UV-Vis absorbance spectra of purified and desalted enzyme without and with addition of NADPH coenzyme (after each 0.5 mM rise in NADPH concentration made in 5 min intervals corresponding blank measurement was performed). The reaction mixture 50 µL enzyme (1.8 mg/mL), 25 mM sodium phosphate buffer, pH 7.5.


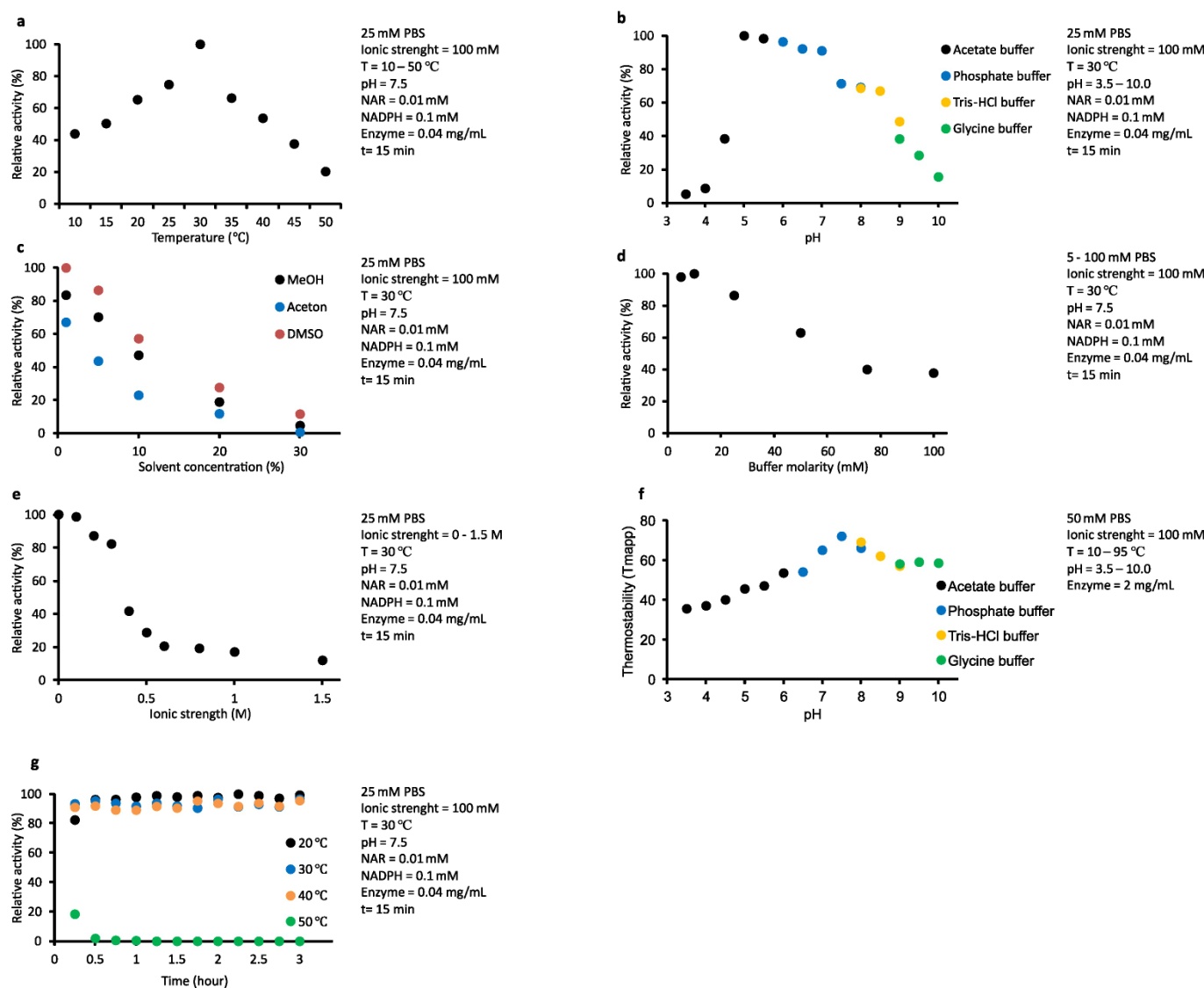


# Supplementary Figure S5. *In vitro* analysis of the biochemical properties of fdeE. Data in all panels represent averages over three replicates. The standard deviations obtained were <3% of relative activity and were not included in the point markers. Effect of (a) temperature, (b) pH, (c) solvent concentration, (d) buffer molarity, (e) ionic strength of fdeE activity. (f) Stability of fdeE analyzed *via* ThermoFAD under different conditions, (g) thermo stability of fdeE.


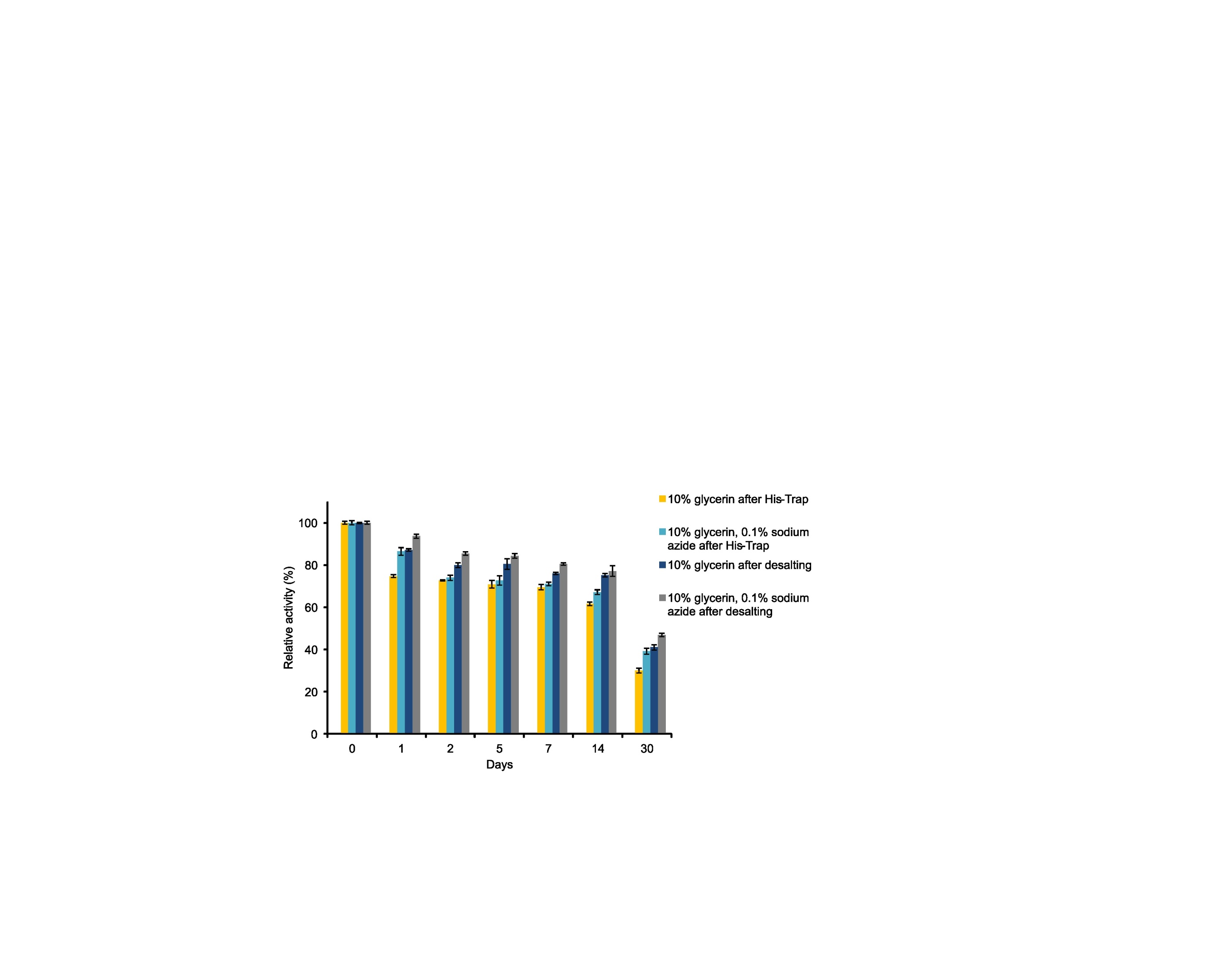


# Supplementary Figure S6. Relative enzymatic activity measured for NADPH consumption in reaction with naringenin after storage at -20 °C purified or desalted protein fraction with 10% glycerol addition and 0.1% addition of sodium azide.

**a.I**

**a.II**

**b.I**

**b.II**

**c.I**

**c.II**

**d.I**

**d.II**

**e.I**

**e.II**

**f.I**

**f.II**

**g.I**

**g.II**

**h.I**

**h.II**

**i.I**

**i.II**

**j.I**

**j.II**

**k.I**

**k.II**

**l.I**

**l.II**

**m.I**

**m.II**

**n.I**

**n.II**

**o.I**

**o.II**

**p.I**

**p.II**

**r.I**

**r.II**

# Supplementary Figure S7. LC-MS analysis of hydroxyl products. Mass spectrum of (a) naringenin, (b) hesperetin, (c) 7-hydroxyflavanone, (d) pinocembrin, (e) eriodictyol, (f) chrysin, (g) baicalein, (h) diosmetin, (i) apigenin, (j) luteolin, (k) 4’,7-dihydroxyflavone, (l) fisetin, (m) quercetin, (n) myricetin, (o) morin, (p) genistein, (r) biochanin A; (I) – substrate, (II) – hydroxy product.

#
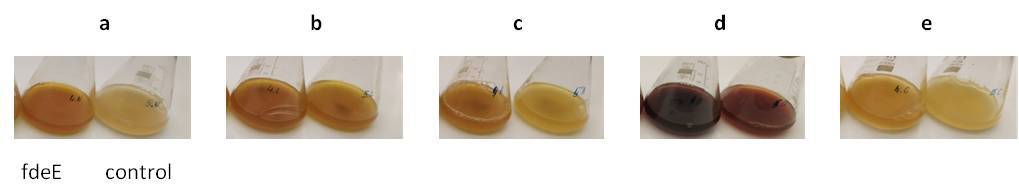


# Supplementary Figure S8. Degradation of C-8 hydroxylation products over time *in vivo* reaction – culture colour after 24 hours. a – naringenin, b – luteolin, c – apigenin, d – quercetin, e – chrysin; left flask – fdeE, right flask– control.


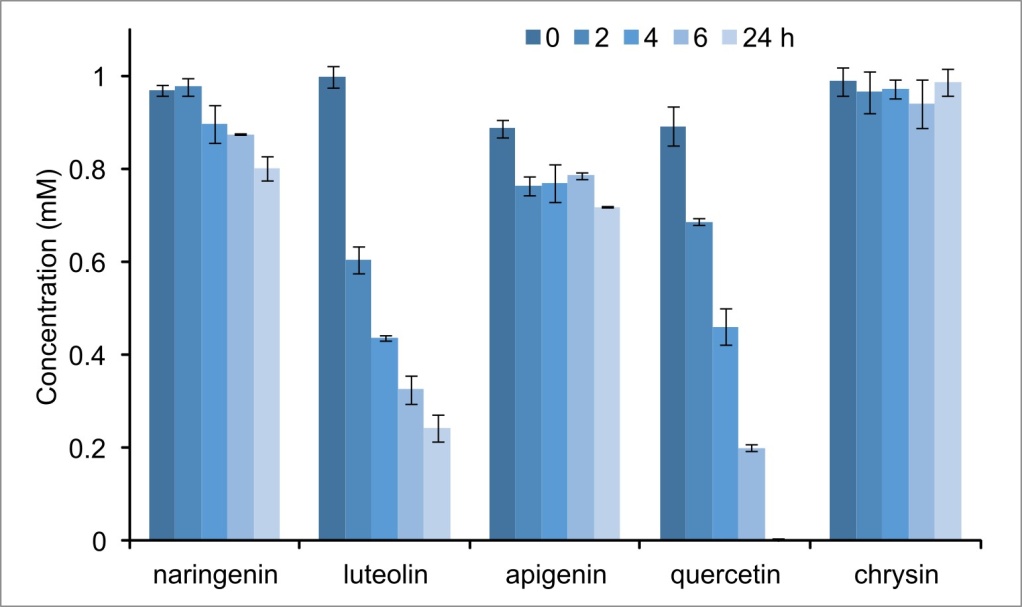


# Supplementary Figure S9. Level of degradation of substrates over time *in vivo* reaction.


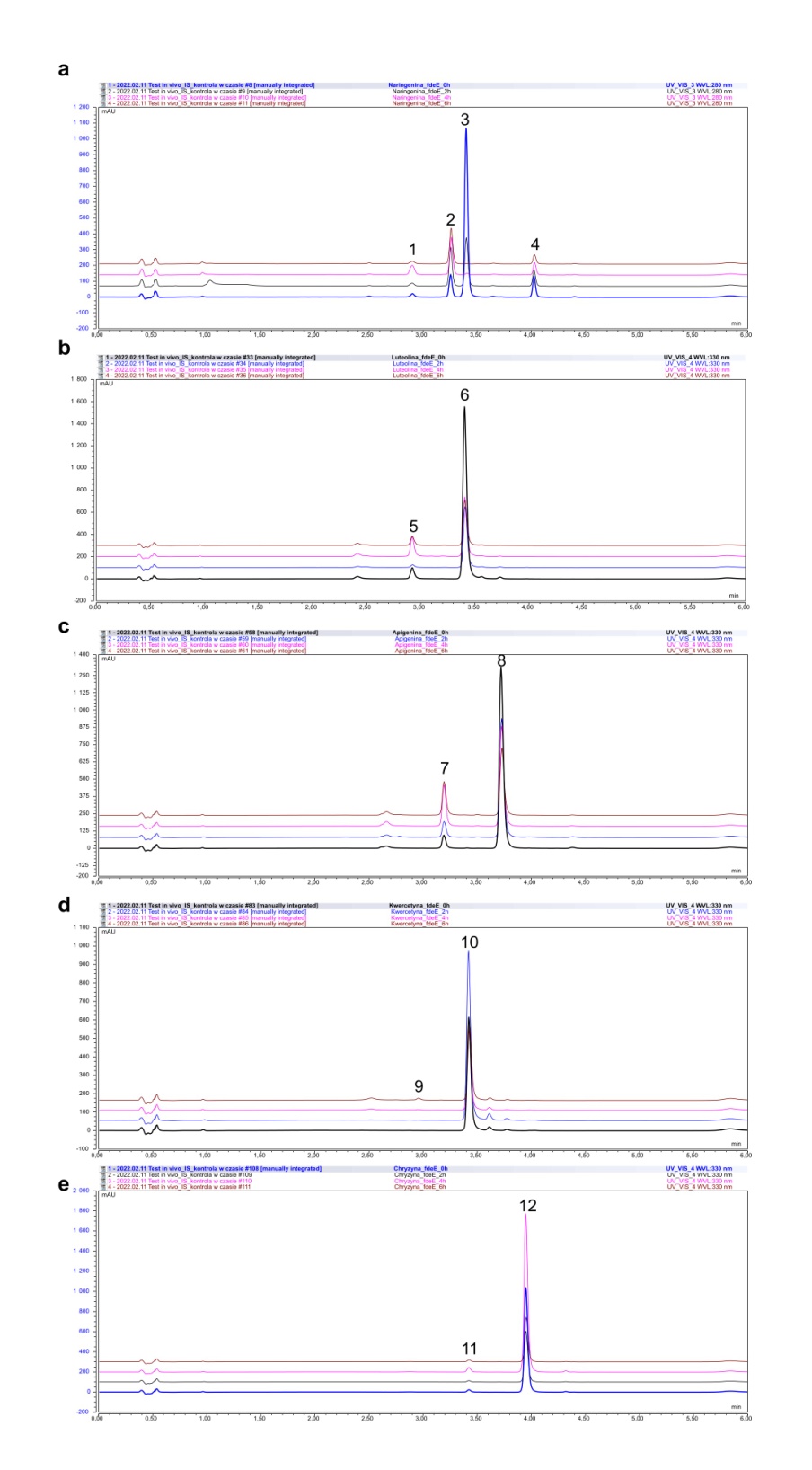


# Supplementary Figure S10. Formation and degradation of C-8 hydroxylation products over time *in vivo* reaction: a – naringenin (λ = 280 nm), b – luteolin (λ = 330 nm), c – apigenin (λ = 330 nm), d – quercetin (λ = 330 nm), e – chrysin (λ = 330 nm); 1 – 8-hydroxynaringenin, 2 – indol (tryptophane degradation product from medium), 3 – naringenin, 4 – dibenzofuran (IS, can be observed at λ = 280 nm, but not at λ = 330 nm), 5 – 8-hydroxyluteolin, 6 – luteolin, 7 – 8-hydroxyapigenin, 8 – apigenin, 9 – 8-hydroxyquercetin, 10 – quercetin, 11 – 8-hydroxychrysin, 12 – chrysin.


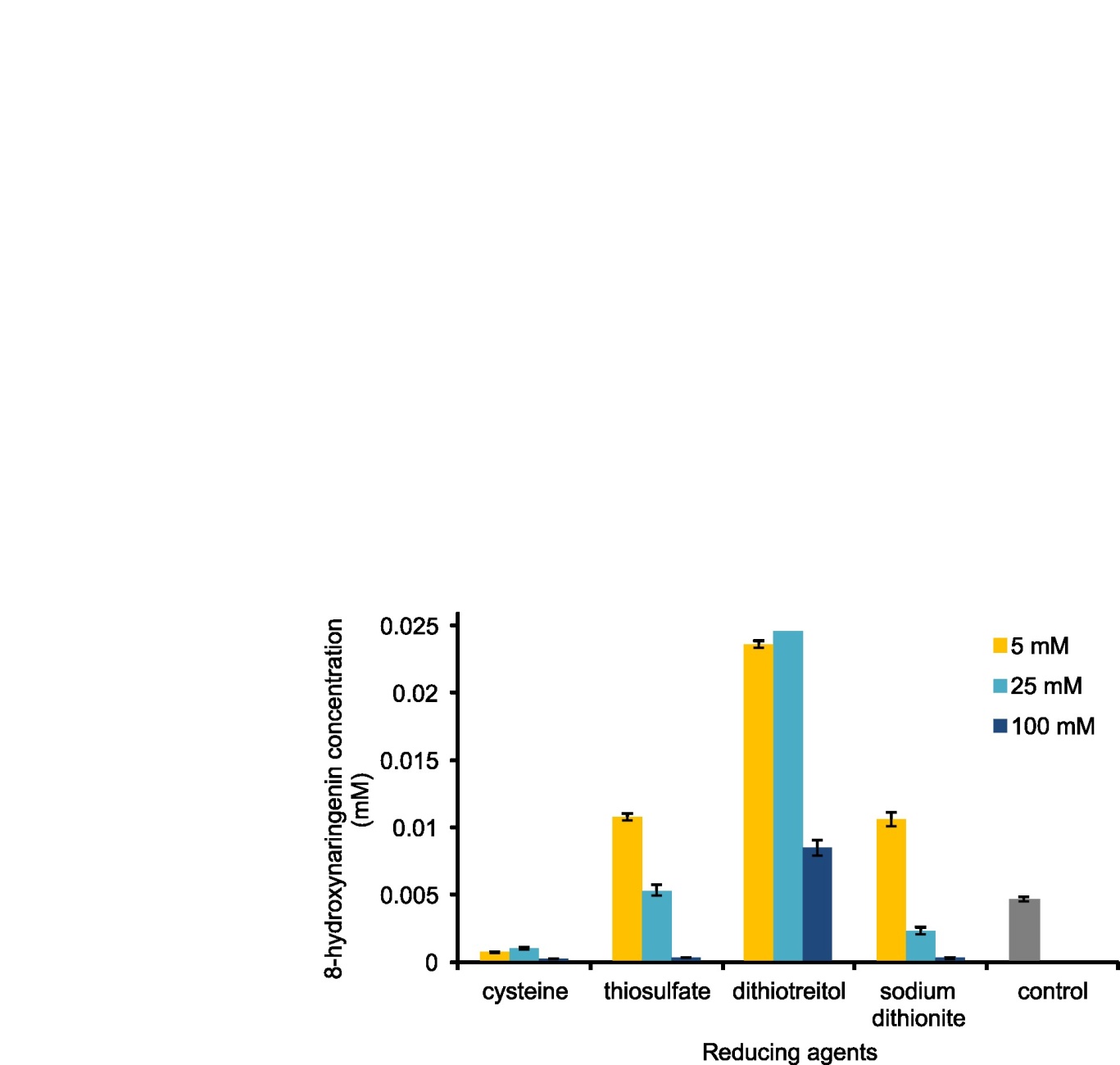


# Supplementary Figure S11. Effect of reducing agents (cysteine, thiosulfate, dithiotreitol, sodium ditionite) on the stability of hydroxy derivatives obtained by fdeE in *vitro* reactions. Naringenin was used as a substrate at an initial concentration of 0.025 mM.


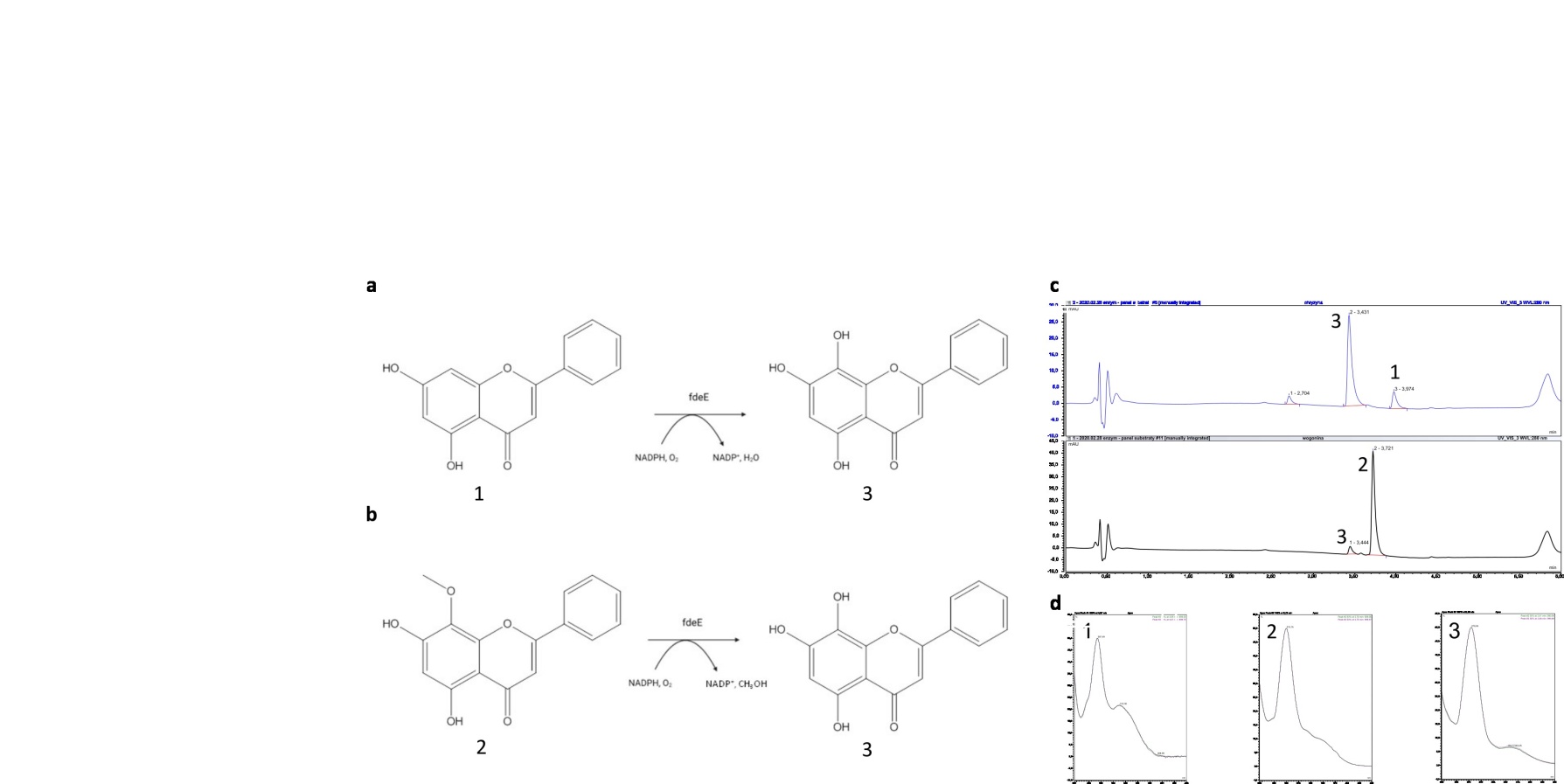


# Supplementary Figure S12. (a) Scheme of the chrysin hydroxylation reaction. (b) Scheme of the wogonin demethylation reaction. (c) UPLC-DAD analysis of samples carried out for chrysin (upper chromatogram) and wogonin (lower chromatogram) using fdeE. (d) UV-Vis absorption spectrum: 1 - chrysin, 2 - wogonin, 3 – 8-hydroxychrysin.


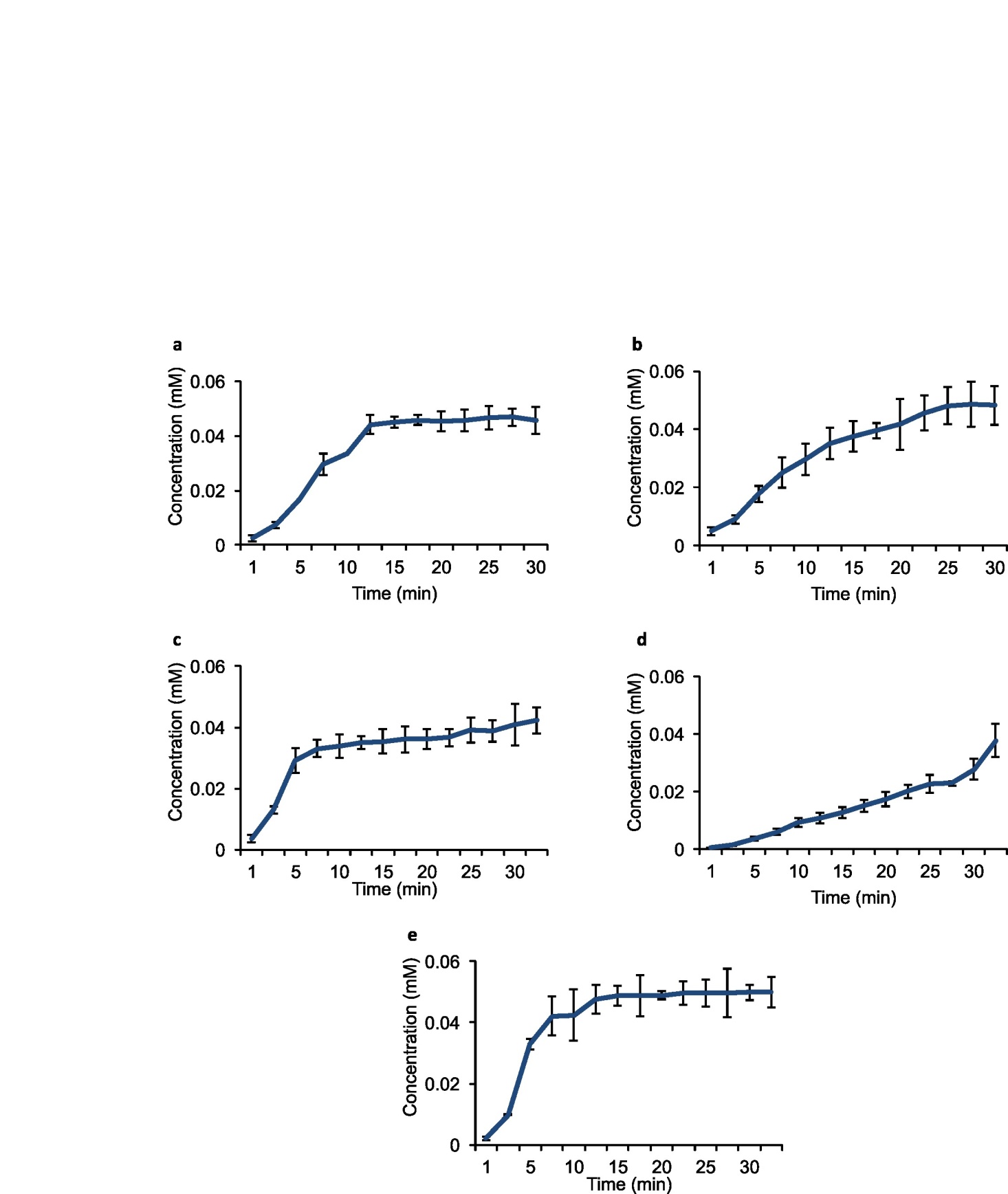


# Supplementary Figure S13. Formation curve of 8-hydroxy derivatives of (a) naringenin, (b) luteolin, (c) apigenin, (d) quercetin, (e) chrysin. The initial substrate concentration was 0.05 mM.


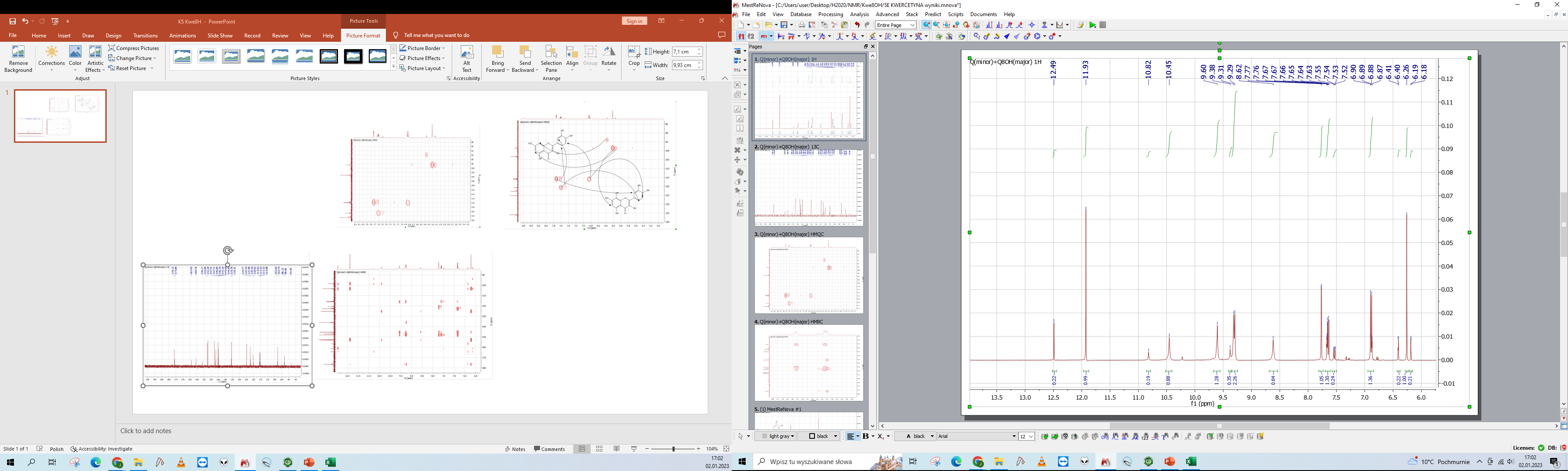


# Supplementary Figure S14. ^1^H-NMR (600 MHz, DMSO-*d_6_*) spectrum of 8-hydroxyquercetin.


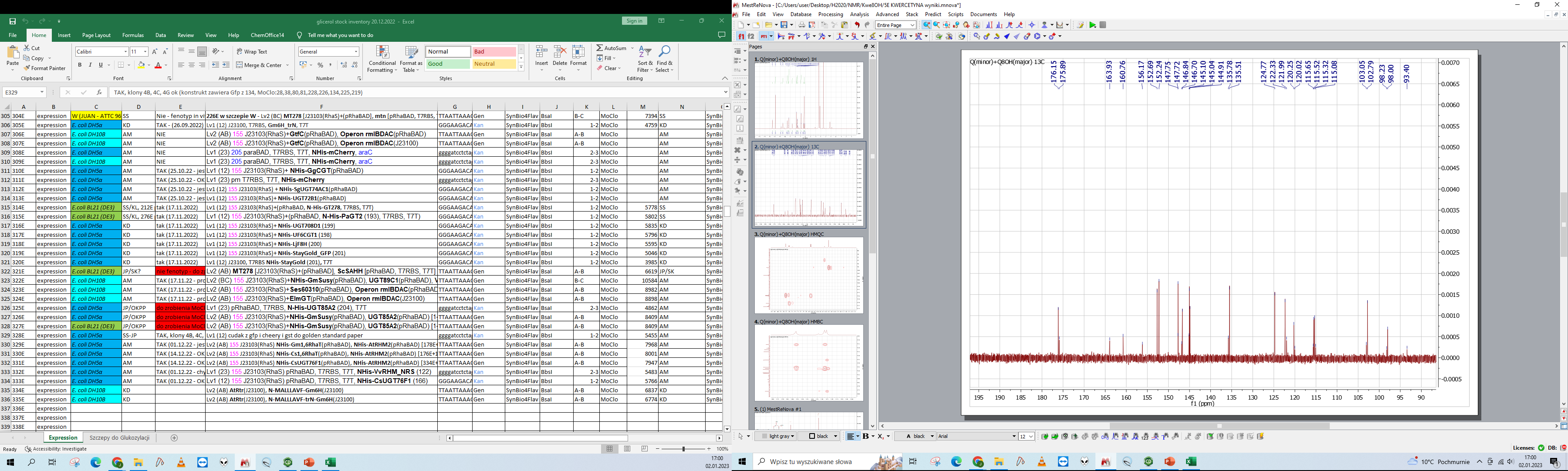


# Supplementary Figure S15. ^13^C-NMR (600 MHz, DMSO-*d_6_*) spectrum of 8-hydroxyquercetin.


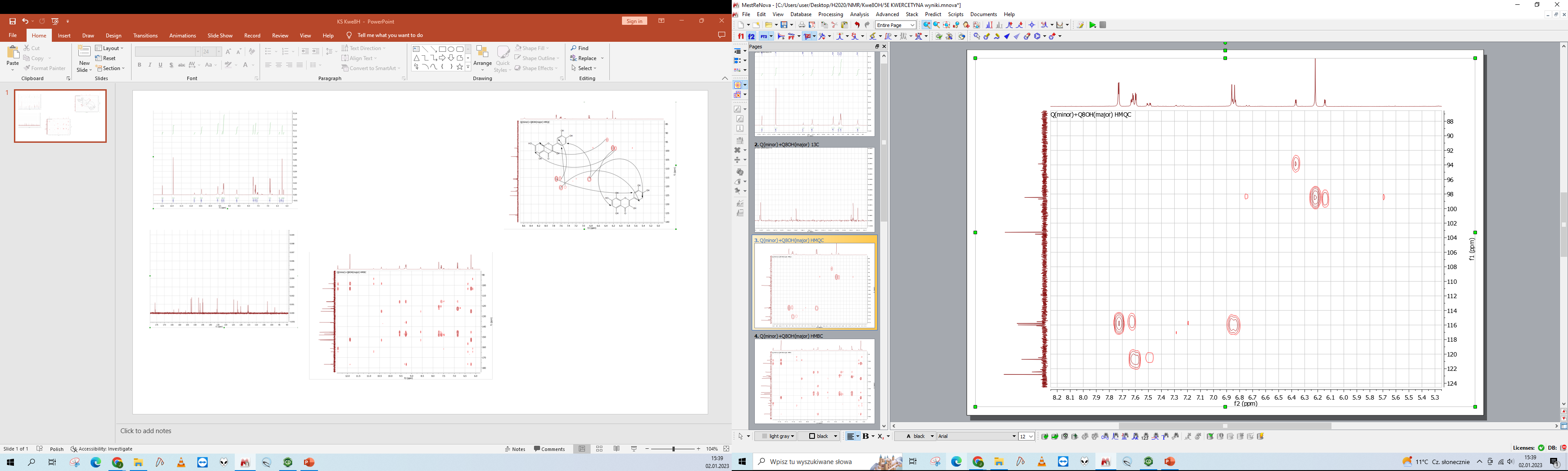


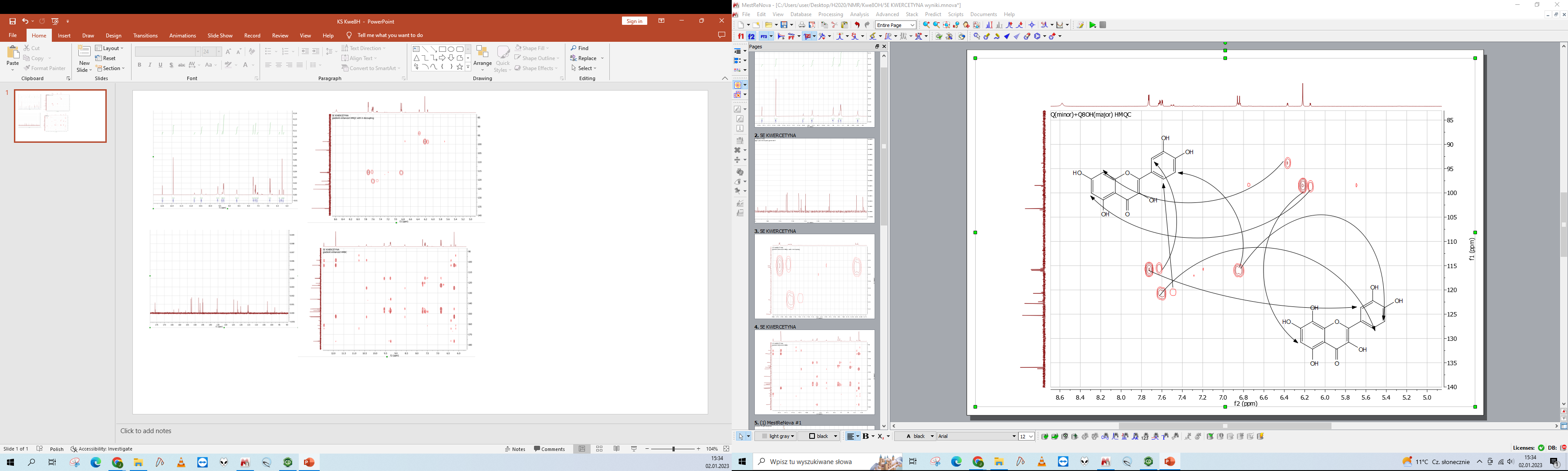


# Supplementary Figure S16. ^1^H-^13^C NMR (HSQC) (600 MHz, DMSO-*d_6_*) spectrum of 8-hydroxyquercetin.

#
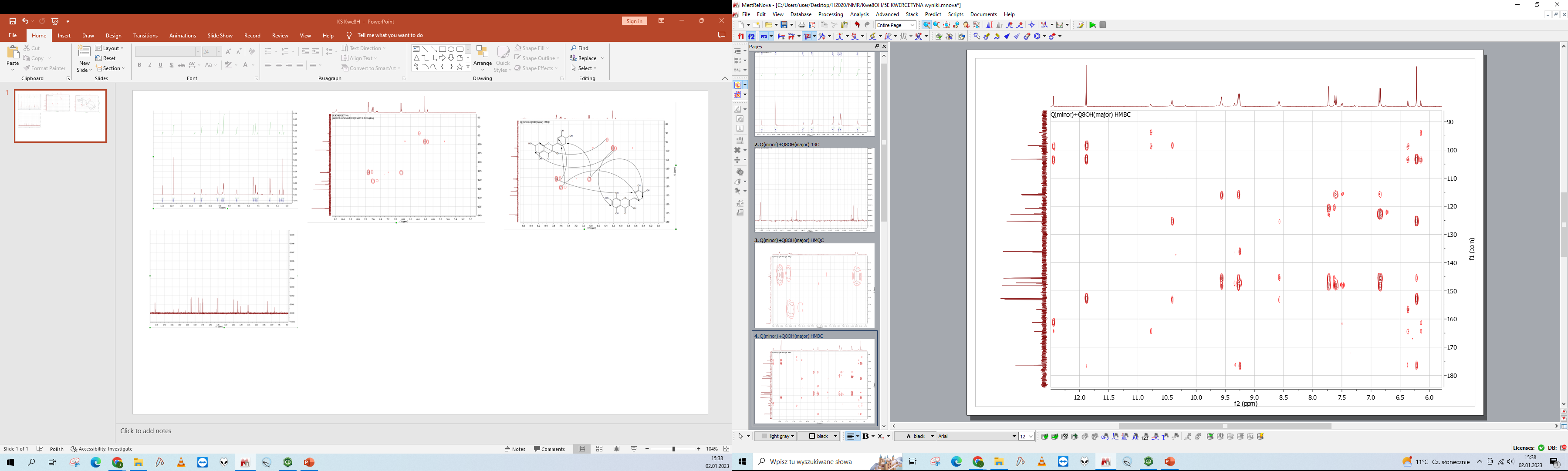


# Supplementary Figure S17. ^1^H-^13^C NMR (HMBC) (600 MHz, DMSO-*d_6_*) spectrum of 8-hydroxyquercetin.

# Supplementary Table S1. Accession numbers and origin of amino acid sequences used in the phylogenetic analysis. A phylogenetic tree was constructed among known flavonoid 6-hydroxylases (F6H)^2–6^, flavonoid 8-hydroxylases (F8H)^5,7–10^ and plant-derived oxygenases capable of flavonoid hydroxylation^10–13^.

| **Name** | **Origin** | **Acc. Number** |
| --- | --- | --- |
| AtF3’H | *Arabidopsis thaliana* | Q9SD85 |
| AtFLS | *Arabidopsis thaliana* | NP_001190266.1 |
| At1g12130 | *Arabidopsis thaliana* | NP_172677.1 |
| At1g12160 | *Arabidopsis thaliana* | NP_172680.1 |
| At1g12200 | *Arabidopsis thaliana* | NP_172684.1 |
| At1g62600 | *Arabidopsis thaliana* | NP_176448.1 |
| CaF6H | *Chrysosplenium americanum* | AAU04696.1 |
| CsF3Ha | *Camellia sinensis* | ASU87419 |
| CsF3Hb | *Camellia sinensis* | ASU87420 |
| CsF8H | *Camellia sinensis* | AAT68774 |
| GhF3’5’H1 | *Gossypium hirsutum* | AAP31058 |
| GmF3’5’H | *Gossypium hirsutum* | AAP31058 |
| GmCYP71D9 | *Glycine max* | NP_001304582.2 |
| GmF3H | *Glycine max* | NP_001236797.1 |
| GmF3’H | *Glycine max* | [AAO47847](https://www.ncbi.nlm.nih.gov/nuccore/AAO47847) |
| GmANS | *Glycine max* | ABY51685.1 |
| GmFLS | *Glycine max* | NP_001237419.1 |
| GeCYP81E1 | *Glycyrrhiza echinata L.* | BAA22422.1 |
| GeCYP93B1 | *Glycyrrhiza echinata L.* | BAA22423.1 |
| GS-OX1 | *Arabidopsis thaliana* | NP_176761.1 |
| GS-OX2 | *Arabidopsis thaliana* | NP_001320894.1 |
| GS-OX3 | *Arabidopsis thaliana* | NP_176444.1 |
| fdeE | *Herbaspirillum seropedicae SmR1* | OQ808967 |
| ItF3’H | *Ipomoea tricolor* | BAD00189 |
| LjF8H | *Lotus japonicus* | BBN79890 |
| MpCYP82D62 | [*Mentha piperita*](https://www.ncbi.nlm.nih.gov/Taxonomy/Browser/wwwtax.cgi?id=34256) | [AGF30366](https://www.ncbi.nlm.nih.gov/protein/451167582) |
| ObCYP93B23 | *Ocimum basilicum* | AGF30365 |
| ObCYP82D33 | *Ocimum basilicum* | [AGF30364](https://www.ncbi.nlm.nih.gov/protein/451167578) |
| ObF8H-1’ | *Ocimum basilicum* | AII16849 |
| ObF8H-2 | *Ocimum basilicum* | AII16848 |
| ObF8H-1 | *Ocimum basilicum* | AII16850 |
| ObPTC52-1 | *Ocimum basilicum* | AII16851.1 |
| OsF3’H | *Oryza sativa* | NP_920627 |
| PhCYP75A1 | *Petunia hybryda* | AUI38391.1 |
| PhCYP75B | *Petunia hybryda* | AAD56282.1 |
| PhF3’5’H1 | *Petunia hybryda* | CAA80265 |
| PhF3’5’H2 | *Petunia hybryda* | CAA80266 |
| RgF8H | *Rhodotorula glutinis KCh735* | OQ808966 |
| Sam5 | *Saccharothrix espanaensis* | ABC88666 |
| SbCYP71D1 | [*Scutellaria baicalensis*](https://www.ncbi.nlm.nih.gov/Taxonomy/Browser/wwwtax.cgi?id=65409) | ASW21048 |
| SbCYP71D2 | *Scutellaria baicalensis* | ASW21049 |
| SbCYP82D1.1 | [*Scutellaria baicalensis*](https://www.ncbi.nlm.nih.gov/Taxonomy/Browser/wwwtax.cgi?id=65409) | ASW21050 |
| SbCYP82D1.2 | *Scutellaria baicalensis* | ASW21051 |
| SbCYP82D2 | *Scutellaria baicalensis* | ASW21052 |
| VvF3’H | *Vitis vinifera* | CAI54277 |
| VvF3’5’H1 | *Vitis vinifera* | CAI54278 |
| YUCCA | *Arabidopsis thaliana* | NP_001329230.1 |
| YUCCA4 | *Arabidopsis thaliana* | NP_196693.1 |
| YUCCA5 | *Arabidopsis thaliana* | NP_199202.1 |
| YUCCA10 | *Arabidopsis thaliana* | NP_175321.1 |

# Supplementary Table S2. Primer sequences and description of transcription units.

| *Primers* | |
| --- | --- |
| **Name** | **Sequence** |
| R24_SEVA182 | AGCGGATAACAATTTCACACAGGA |
| F24_SEVA182 | CGCCAGGGTTTTCCCAGTCACGAC |
| rhaB_expression module_Fwd | GGCGCTTTTTAGACTGGTCG |
| rha S middle F | GGACGGGATGGCTTTCTGCAATAA |

| *Transcription units* | | | |
| --- | --- | --- | --- |
| **Plasmid** | **Characteristics** | **Strain** | **Used for** |
| Lv1 fdeE | Km^R^, pBBR1, Lv1 pSEVA23g19g1 vector, pJ23100, RBS T7 from pET28, fdeE gene, and T7 terminator from pET28 | *E. coli* DH5α | *In vivo* assay |
| Lv1 N-His-fdeE | Km^R^, pBBR1, Lv1 pSEVA23g19g1 vector containing RhaS gene, RhaBAD promoter, RBS T7 from pET28, fdeE gene fussion with N-terminal His tag, and T7 terminator from pET28 | *E. coli* BL21 (DE3) | Overexpression analysis/ Formation rate/ Stabilization assay |
| Lv1 N-His-RgF8H | Dulak et al., 2022 | *E. coli* BL21(DE3) | Formation rate |
| Lv1 N-His-Bm_GDH | Dulak et al., 2022 | *E. coli* BL21 (DE3) | Stabilization assay |

#

# Supplementary Table S3. Structures of substrates used in this work. The shaded boxes in colour represent compounds transformed by fdeE.

| **Compounds related to this work** | **Structure** | **Compounds related to this work** | **Structure** |
| --- | --- | --- | --- |
| Naringenin |  | Hesperetin |  |
| 2’-hydroxyflavanone |  | 3’-hydroxyflavanone |  |
| 4’-hydroxyflavanone |  | 6-hydroxyflavanone |  |
| 7-hydroxyflavanone |  | Isoxanthohumol |  |
| Pinocembrin |  | Eriodictyol |  |
| Chrysin |  | Baicalein |  |
| Diosmetin |  | Apigenin |  |
| Luteolin |  | 3-hydroxyflavone |  |
| 4’,7-dihydroxyflavone |  | Wogonin |  |
| Quercetin |  | Myricetin |  |
| Morin |  | Fisetin |  |
| Epicatechin |  | Dihydromyricetin |  |
| Biochanin A |  | Daidzein |  |
| Genistein |  | α,β-dihydroxanthohumol |  |
| Phloretin |  | Resveratrol |  |
| Xanthohumol |  | 4’-hydroxychalcone |  |
| Mequinol |  | 4-phenylphenol |  |
| 4-hydroxybenzaldehyde |  | Rezorcin |  |
| 4-isopropylphenol |  | Pirocatechin |  |
| o-cresol |  | 1-naphthal |  |
| 2,4-dinitrophenol |  | p-hydroxyacetophenone |  |
| Vanillin |  | o-nitrophenol |  |
| m-nitrophenol |  | Phenol |  |
| Hydroquinone |  | Guaiacol |  |
| 4-ethylphenol |  | 2,4-dihydroxyacetophenone |  |

#

# Supplementary Table S4. Calculated parameters of substrate binding pocket volume for the predicted 3D models of fdeE, RgF8H and LjF8H.

| **Enzyme** | **Volume** | **Samples** |
| --- | --- | --- |
| RgF8H | 2159.7 | 40100 |
| LjF8H | 1102.8 | 40100 |
| fdeE | 3031.4 | 40100 |

# Supplementary Table S5. Reaction yield and purification efficiencies. The yield of the isolated product following the best purification method is 16%. Theoretical total amount calculated for the entire reaction mixture (17.1 mg) based on conversion percentage and then divided into 3 fractions.

|  | 8-hydroxyquercetin (mg) | Isolated reaction yield  (%) | Purification efficiency (%) |
| --- | --- | --- | --- |
| Theoretical Total Amount | 5.7 | - | - |
| Ethyl Acetate | 5.16 | 16.33 | 90.58 |
| 1-Buthanol | 2.6 | 8.23 | 45.61 |
| Liophilization | 0.48 | 1.52 | 8.42 |

# Products identification

Products were identified by retention time, UV spectrum, and mass analysis.

Flavones: 8-hydroxychrysin (calculated mass for the molecular formula C15H10O5 for [M+H]+ m/z+ ~ 271.24, [M-H]- m/z- ~ 269.24, for which the observed masses are 271 and 269, respectively, λmax: 280 nm, 26.93 U (micromoles of substrate converts per minute per milligram of enzyme)), 8-hydroxybaicalein (calculated mass for the molecular formula C15H10O6 for [M+H]+ m/z+ ~ 287.24, [M-H]- m/z- ~ 285.24, for which the observed masses are 287 and 285, respectively, λmax: 286 nm, 6.18 U), 8-hydroxydiosmetin (calculated mass for the molecular formula C16H12O7 for [M+H]+ m/z+ ~ 317.07, [M-H]- m/z- ~ 315.07, for which the observed masses are 317 and 315, respectively, λmax: 280 and 336 nm, 24.95 U), 8-hydroxyapigenin (calculated mass for the molecular formula C15H10O6 for [M+H]+ m/z+ ~ 289.06, [M-H]- m/z- ~ 287.06, for which the observed masses are 289 and 287, respectively, λmax: 280 and 303 nm, 41.22 U), of 8-hydroxyluteolin (calculated mass for the molecular formula C15H10O7 for [M+H]+ m/z+ ~ 303.24, [M-H]- m/z- ~ 301.24, for which the observed masses are 303 and 301, respectively, λmax: 280 and 337 nm, 15.96 U), 7,8,4’-trihydroxyflavone (calculated mass for the molecular formula C15H10O5 for [M+H]+ m/z+ ~ 271.24, [M-H]- m/z- ~ 269.24, for which the observed masses are 271 and 269, respectively, λmax: 265 and 327 nm, 4.81 U) were obtained using fdeE.

Flavanones: 8-hydroxynaringenin (calculated mass for the molecular formula C15H12O6 for [M+H]+ m/z+ ~ 289.25, [M-H]- m/z- ~ 287.25, for which the observed masses are 289 and 287, respectively, λmax: 295 nm, 38.41 U), 8-hydroxyhesperetin (calculated mass for the molecular formula C16H14O7 for [M+H]+ m/z+ ~ 319.28, [M-H]- m/z- ~ 317.28, for which the observed masses are 319 and 317, respectively, λmax: 292 nm, 38.45 U), 7,8-dihydroxyflavanone (calculated mass for the molecular formula C15H12O4 for [M+H]+ m/z+ ~ 257.25, [M-H]- m/z- ~ 255.25, for which the observed masses are 257 and 255, respectively, λmax: 292 nm, 44.52 U), 8-hydroxypinocembrin (calculated mass for the molecular formula C15H12O5 for [M+H]+ m/z+ ~ 273.25, [M-H]- m/z- ~ 271.25, for which the observed masses are 273 and 271, respectively, λmax: 295 nm, 26.95 U), 8-hydroxyeriodictyol (calculated mass for the molecular formula C15H12O7 for [M+H]+ m/z+ ~ 305.25, [M-H]- m/z- ~ 303.25, for which the observed masses are 305 and 303, respectively, λmax: 292 nm, 35.03 U), using fdeE.

Flavonoles: 8-hydroxyfisetin (calculated mass for the molecular formula C15H10O7 for [M+H]+ m/z+ ~ 303.24, [M-H]- m/z- ~ 301.24, for which the observed masses are 303 and 301, respectively, λmax: 255 and 363 nm, 21.51 U), 8-hydroxyquercetin (calculated mass for the molecular formula C15H10O8 for [M+H]+ m/z+ ~ 319.24, [M-H]- m/z- ~ 317.24, for which the observed masses are 319 and 317, respectively, λmax: 260 and 339 nm, 22.51 U), 8-hydroxymyricetin (calculated mass for the molecular formula C15H10O9 for [M+H]+ m/z+ ~ 335.24, [M-H]- m/z- ~ 333.24, for which the observed masses are 335 and 333, respectively, λmax: 258 and 341 nm, 20.66 U), 8-hydroxymorin (calculated mass for the molecular formula C15H10O8 for [M+H]+ m/z+ ~ 319.24, [M-H]- m/z- ~ 317.24, for which the observed masses are 319 and 317, respectively, λmax: 255 and 354 nm, 45.59 U) obtained with fdeE.

Isoflavones: 8-hydroxygenistein (calculated mass for the molecular formula C15H10O6 for [M+H]+ m/z+ ~ 287.24, [M-H]- m/z- ~ 285.24, for which the observed masses are 287 and 285, respectively, λmax: 267 nm, 7.90 U), and 8-hydroxybiochanin A (calculated mass for the molecular formula C16H12O6 for [M+H]+ m/z+ ~ 301.26, [M-H]- m/z- ~ 299.26, for which the observed masses are 301 and 299, respectively, λmax: 267 nm, 1.88 U) by fdeE.

8-hydroxyquercetin: 1H NMR (600 MHz, DMSO-d6) δ (ppm): 6.22 (1H, s, H-6), 6.89 (1H, d, J = 8.4 Hz, H-5’), 7.65 (1H, dd, J = 2.1; 8.4 Hz, H-2’), 7.77 (1H, d, J = 2.1 Hz, H-6’), 8.62 (1H, s, 8-OH), 9.29 (1H, s, 3-OH), 9.31 (1H, s, 3’-OH), 9.60 (1H, s, 4’-OH), 10.45 (1H, s, 7-OH), 11.93 (1H, s, 5-OH); 13C NMR (150 MHz, DMSO-d6) δ (ppm): 98.0 (C-6), 102.79 (C-10), 115.32 (C-6’), 115.52 (C-5’), 120.25 (C-2’), 122.33 (C-1’), 124.77 (C-8), 135.78 (C-3), 144.91 (C-9), 145.04 (C-3’), 146.7 (C-2), 147.72 (C-4’), 152.24 (C-5), 152.69 (C-7), 176.15 (C-4).

References

1. Jurcik, A. *et al.* CAVER Analyst 2.0: analysis and visualization of channels and tunnels in protein structures and molecular dynamics trajectories. *Bioinformatics* **34**, 3586–3588 (2018).

2. Anzellotti, D. & Ibrahim, R. K. Novel flavonol 2-oxoglutarate dependent dioxygenase: affinity purification, characterization, and kinetic properties. *Archives of Biochemistry and Biophysics* **382**, 161–172 (2000).

3. Latunde-Dada, A. O. *et al.* Flavonoid 6-hydroxylase from soybean (*Glycine max L.*), a novel plant P-450 monooxygenase. *Journal of Biological Chemistry* **276**, 1688–1695 (2001).

4. Anzellotti, D. & Ibrahim, R. K. Molecular characterization and functional expression of flavonol 6-hydroxylase. *BMC Plant Biology* **4**, 20 (2004).

5. Zhao, Q. *et al.* Two CYP82D enzymes function as flavone hydroxylases in the biosynthesis of root-specific 4′-deoxyflavones in *Scutellaria baicalensis*. *Molecular Plant* **11**, 135–148 (2018).

6. Berim, A. & Gang, D. R. The roles of a flavone-6-hydroxylase and 7-*O*-demethylation in the flavone biosynthetic network of sweet basil. *Journal of Biological Chemistry* **288**, 1795–1805 (2013).

7. Dulak, K. *et al.* Novel flavonoid C-8 hydroxylase from *Rhodotorula glutinis*: identification, characterization and substrate scope. *Microbial Cell Factories* **21**, 175 (2022).

8. Marin, A. M. *et al.* Genetic and functional characterization of a novel *meta*-pathway for degradation of naringenin in *Herbaspirillum seropedicae* SmR1. *Environmental Microbiology* **18**, 4653–4661 (2016).

9. Hiraga, Y. *et al.* Identification of a flavin monooxygenase-like flavonoid 8-hydroxylase with gossypetin synthase activity from *Lotus japonicus*. *Plant and Cell Physiology* **62**, 411–423 (2021).

10. Berim, A., Park, J.-J. & Gang, D. R. Unexpected roles for ancient proteins: flavone 8-hydroxylase in sweet basil trichomes is a Rieske-type, PAO-family oxygenase. *Plant Journal* **80**, 385–395 (2014).

11. Hansen, B. G., Kliebenstein, D. J. & Halkier, B. A. Identification of a flavin-monooxygenase as the *S*-oxygenating enzyme in aliphatic glucosinolate biosynthesis in *Arabidopsis*. *The Plant Journal* **50**, 902–910 (2007).

12. Mizutani, M. & Ohta, D. Diversification of P450 genes during land plant evolution. *Annual Review of Plant Biology* **61**, 291–315 (2010).

13. Kawai, Y., Ono, E. & Mizutani, M. Evolution and diversity of the 2–oxoglutarate-dependent dioxygenase superfamily in plants. *The Plant Journal* **78**, 328–343 (2014).
